# Protein phosphatase EYA1 regulates the dephosphorylation and turnover of BCL2L12 to promote glioma development

**DOI:** 10.7150/ijbs.99619

**Published:** 2025-01-13

**Authors:** Tianzi Wei, Risheng Lin, Yi Lu, Dong-Yan Jin, Jian Zhang, Mai Har Sham

**Affiliations:** 1 School of Biomedical Sciences, Li Ka Shing Faculty of Medicine, The University of Hong Kong, Pokfulam, Hong Kong SAR, China.; 2 Department of Biology, School of Life Sciences, Southern University of Science and Technology, Shenzhen, Guangdong, China.; 3 School of Medicine, Southern University of Science and Technology, Shenzhen, Guangdong, China.; 4 Joint Laboratory of Guangdong-Hong Kong Universities for Vascular Homeostasis and Diseases, School of Medicine, Southern University of Science and Technology, Shenzhen, Guangdong, China.; 5 School of Biomedical Sciences, Faculty of Medicine, The Chinese University of Hong Kong, Shatin, Hong Kong SAR, China.

**Keywords:** EYA1, BCL2L12, Phosphatase, Glioma

## Abstract

Glioma is the most prevalent and deadly type of intracranial tumor. Understanding the molecular drivers and their underlying mechanisms in glioma development is urgently needed. EYA1 is a unique protein phosphatase that drives gliomagenesis, yet its substrates remain largely uncharacterized. In this study, we identify BCL2L12 (BCL2-like 12), a critical oncoprotein in glioma, as a novel substrate of EYA1 phosphatase in glioma cells. Our findings demonstrate that EYA1 dephosphorylates BCL2L12 at threonine-33 (T33), which in turn protects BCL2L12 from ubiquitination and subsequent proteasomal degradation. Our results indicate that BCL2L12 partially mediates the oncogenic roles of EYA1 in promoting glioma cell proliferation, highlighting the significance of EYA1's dephosphorylation of BCL2L12 in tumor progression. Moreover, we validate a positive correlation between EYA1 and BCL2L12 protein levels in glioma patient samples. In summary, our study reveals how EYA1-BCL2L12 interaction functions in glioma development, implicating EYA1 as a potential therapeutic target for glioma treatment.

## Introduction

Glioma is the most common intracranial tumor, representing 78.3% of all primary malignant tumors in the central nervous system 1. Despite the widespread use of surgery, radiotherapy, and chemotherapy, glioma patients still have a poor prognosis 2-4. Understanding the molecular factors that drive glioma formation and progression is a key focus in glioma research.

The eyes absent (EYA) gene family, comprising EYA1-4, encodes a group of protein phosphatases crucial for mammalian embryo development, including craniofacial morphogenesis, organogenesis of bone, cartilage, inner ear, kidney, and thymus 5-11. Recent studies reveal that EYAs are dysregulated in various brain tumors and play a key role in brain cancer development, including glioma, medulloblastoma, and neuroblastoma. In comparison to healthy tissues, the expression levels of EYAs are elevated in glioma tumors 12-14. EYA1 is required for the maintenance of stemness and survival of glioblastoma stem cells (GSCs) 15. Additionally, EYA2 phosphatase inhibition has been shown to prevent gliomagenesis and medulloblastoma progression 16-18. However, the molecular mechanisms by which EYAs regulate glioma development still require further investigation.

EYAs, as protein phosphatases, remove phosphate groups from tyrosine or threonine residues in their substrates 19-22. Dephosphorylation by EYAs significantly impacts various biological processes. For example, EYAs dephosphorylate the tyrosine-142 (Y142) on the histone H2AX in response to DNA damage, promoting DNA repair rather than apoptosis 23, 24. EYA1/4 dephosphorylate the tyrosine 445 (Y445) on PLK1 in the G2 phase to promote centrosome maturation in cell division 25. Importantly, dephosphorylation by EYAs influences the turnover of their substrates. For instance, the dephosphorylation of threonine-58 (T58) on c-Myc by EYAs prevents it from ubiquitination, which enhances c-Myc stability in nephrogenesis and breast carcinogenesis 8, 26. In our previous study, we show that EYA1 dephosphorylates threonine-2122 (T2122) on Notch1, which promotes Notch1 protein stability and maintains Notch signaling activity to regulate neurogenesis in mammalian craniofacial morphogenesis 27, 28. Notably, both c-Myc and Notch1 have a conserved “TPXXSP” motif where EYA1 dephosphorylates the threonine (T) in this motif 27. However, the specific substrates and molecular functions of EYA phosphatases in glioma development remain largely uncharacterized.

In this study, we employed an unbiased phosphoproteomic approach and identified the essential glioma oncoprotein BCL2L12 as a novel substrate of EYA1 in glioma cells. We demonstrate that BCL2L12 interacts and colocalizes with EYA1 in glioma cells. EYA1 dephosphorylates threonine-33 (T33) on BCL2L12, thereby stabilizing BCL2L12 by preventing its ubiquitination and subsequent proteasome-mediated degradation. Additionally, we show that BCL2L12 partially mediates the oncogenic function of EYA1 in glioma cells in a dephosphorylation-dependent manner. Furthermore, we validated the regulatory relationship between EYA1 and BCL2L12 in glioma by examining protein expression in patient samples. Our study reveals that EYA1 controls the turnover of BCL2L12 by regulating BCL2L12 dephosphorylation and stabilization, implicating EYA1 as a potential therapeutic target for glioma treatment.

## Results

### BCL2L12 is a potential substrate of EYA1 in glioma cells

To analyze EYA1 expression pattern across various tumors, we first employed Tumor Immune Single Cell Hub (TISCH), a database collecting 2,045,746 cells from 28 types of human cancers 29. Our analysis revealed that EYA1 was remarkably overexpressed in glioma malignant cells, unlike in other cell types and cancers (Figure S1A-C). To select an appropriate glioma cell model, we assessed EYA1 expression levels across multiple glioma cell lines from the Cancer Cell Line Encyclopedia (CCLE) 30. We selected T98G, U87MG, and U251MG for subsequent phenotypic and mechanistic studies based on their varying EYA1 expression levels: high, medium, and low, respectively (Figure S2A).

**Figure 1:**
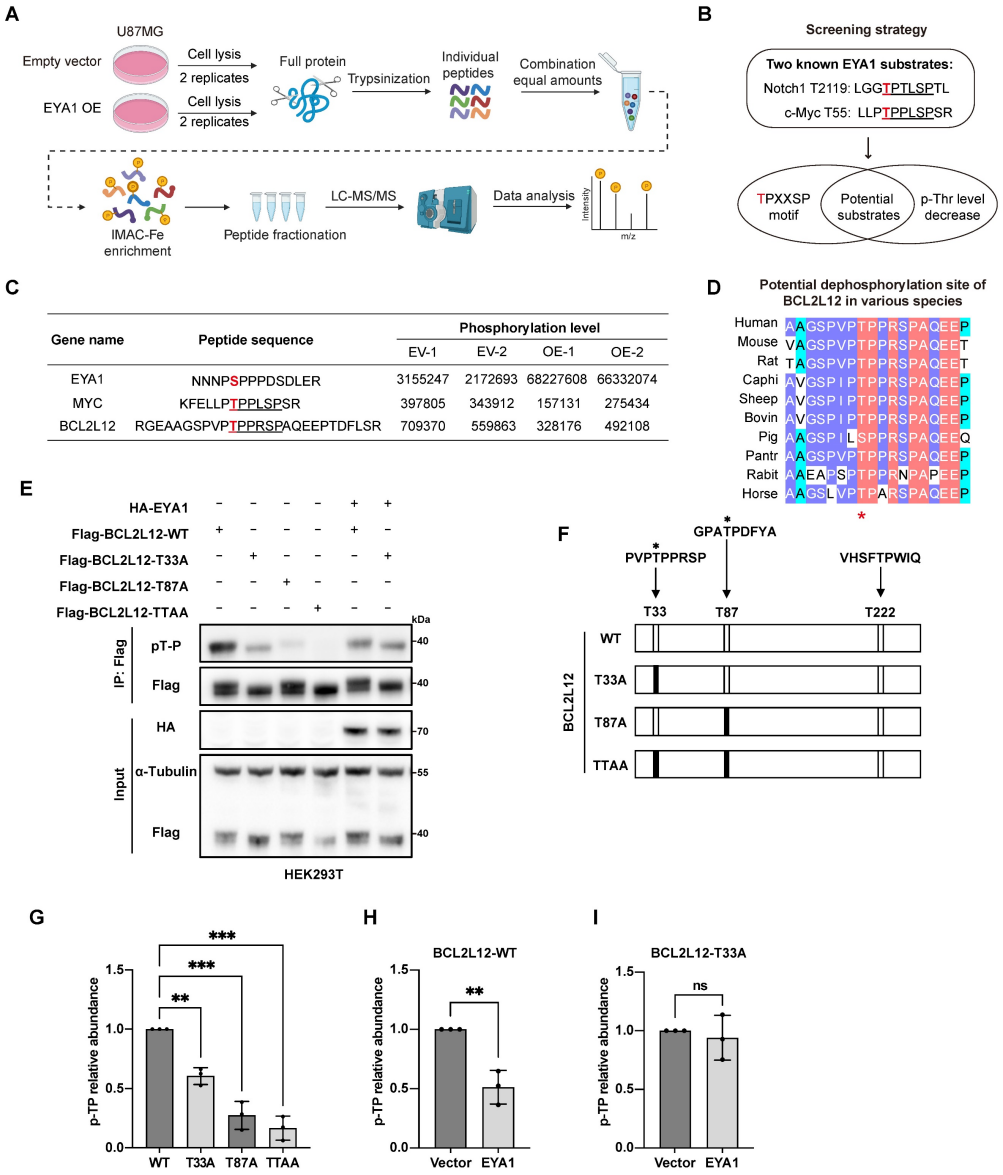
** EYA1 potentially dephosphorylates BCL2L12 at T33 in glioma cells.** A, Scheme flow chart of phosphoproteomic analysis. B, Criteria for searching novel substrate of EYA1 in glioma cells. C, Table showing the changes of phosphorylation level of representative peptides in response to EYA1 overexpression. Phosphorylation sites were highlighted with red color. “TPXXSP” motif is highlighted with black underline. D, Sequence alignment of potential EYA1-regulated region in BCL2L12 among various species. E, T33 was a potential dephosphorylation site of BCL2L12 by EYA1. HEK293T cells were transfected with wild-type or various phospho-dead mimic mutations of BCL2L12. Cells were lysed and subjected to IP using anti-flag beads and immunoblotting using p-TP specific antibody. F, Schematic diagram of BCL2L12 “T-P” dipeptide and phosphor-dead mimic mutants. G-I, Quantitative statistic results of the phosphorylation level of wild-type and phospho-dead mimic mutations of BCL2L12 with or without EYA1 overexpression. *P < 0.05, **P < 0.01, ***P < 0.001, ns, no significance.

To determine substrates of EYA1 threonine phosphatase in glioma, we stably overexpressed EYA1 in U87MG cells and conducted phosphoproteomic analysis (Figure 1A and Figure S2B). This analysis identified 15,777 modified peptides from 4,032 proteins (Supplementary Data). Among 12,706 modified sites, 820 showed significant changes in phosphorylation levels in response to EYA1 overexpression (|Fold change| > 1.5 and P < 0.05). Among these, 478 were upregulated, and 342 were downregulated in EYA1 overexpressing cells. Enrichment analysis of these differential phosphorylated proteins indicated that EYA1 overexpression impacted diverse signaling factors related to cell growth, including chromatin organization and cell cycle signaling pathway (Figure S3A-C).

Notch1 and c-Myc are well-established targets regulated by EYA1's threonine phosphatase activity. Both proteins contain a conserved “TPXXSP” motif, where EYA1 dephosphorylates the threonine residue 27. Building on this finding, we screened for potential substrates of EYA1 in glioma cells using two criteria: the presence of the conserved “TPXXSP” motif; and the threonine phosphorylation level in this motif should be decreased in response to EYA1 overexpression (Figure 1B). We found that, besides the well-established c-Myc, only BCL2L12 met both criteria in our phosphoproteome data (Figure 1C and Figure S4), suggesting that BCL2L12 might be a potential EYA1 substrate in glioma cells. Interestingly, the predicted 3D structures of Notch1, c-Myc, and BCL2L12 showed that the potential dephosphorylation sites targeted by EYA1 were found in the flexible loop region, which is exposed to the external environment, making these sites accessible for dephosphorylation (Figure S5A-C). Given that c-Myc is a known EYA1 substrate in other biological contexts, and BCL2L12 is an essential oncoprotein in glioma development, we decided to focus our further investigations on BCL2L12.

Based on our phosphoproteomic data, we predict that EYA1 may dephosphorylate the threonine-33 (T33) of BCL2L12, which is highly conserved across various species (Figure 1D). To test this hypothesis, we employed phosphatase assays using an anti-phospho-threonine-proline antibody that specifically recognizes phosphorylated threonine with a neighboring proline (pT-P) in a peptide 27. Currently, there is no commercially available antibody that specifically recognizes phosphorylated BCL2L12. Since T33 is adjacent to proline (P34) at the carboxyl end of BCL2L12, we hypothesized that the anti-phospho-threonine-proline antibody, previously used to confirm T2122 as a dephosphorylation site on Notch1 27, could also be used to detect the phosphorylation level of T33 on BCL2L12. There are three threonine-proline dipeptides (T33P, T87P, and T222P) in the primary sequence of BCL2L12, and two of them (T33 and T87) can be phosphorylated, as previously reported 31 (Figure 1F). To validate EYA1's dephosphorylation of BCL2L12 at T33, we generated several phospho-dead mutants (T33A, T87A, and the double site mutation TTAA) that represent the unphosphorylated forms (Figure 1F).

Flag-BCL2L12, either wild-type or mutated, was transfected into HEK293T cells, both with and without HA-EYA1. The cell lysates were then analyzed using an anti-Flag antibody to detect total BCL2L12 and an anti-pT-P antibody to assess phosphorylated BCL2L12. Consequently, a notable reduction in BCL2L12 phosphorylation was observed in the T33A, T87A, and TTAA mutants when EYA1 was not overexpressed, indicating the effective performance of the anti-pT-P antibody (Figure 1E and G). Interestingly, the reduction in phosphorylation of the T87A mutant was greater than that of the T33A mutant (Figure 1E and G). This suggests that the phosphorylation status of T33 is affected by the phosphorylation level of T87; when T87 is not phosphorylated, T33 remains relatively unphosphorylated. Furthermore, EYA1 overexpression significantly reduced phosphorylation of wild-type BCL2L12 (Figure 1E and H). However, this reduction was blocked by the T33A mutation (Figure 1E and I), indicating that T33 may serve as a potential dephosphorylation site for EYA1 on BCL2L12.

### EYA1 interacts and colocalizes with BCL2L12 in glioma cells

**Figure 2:**
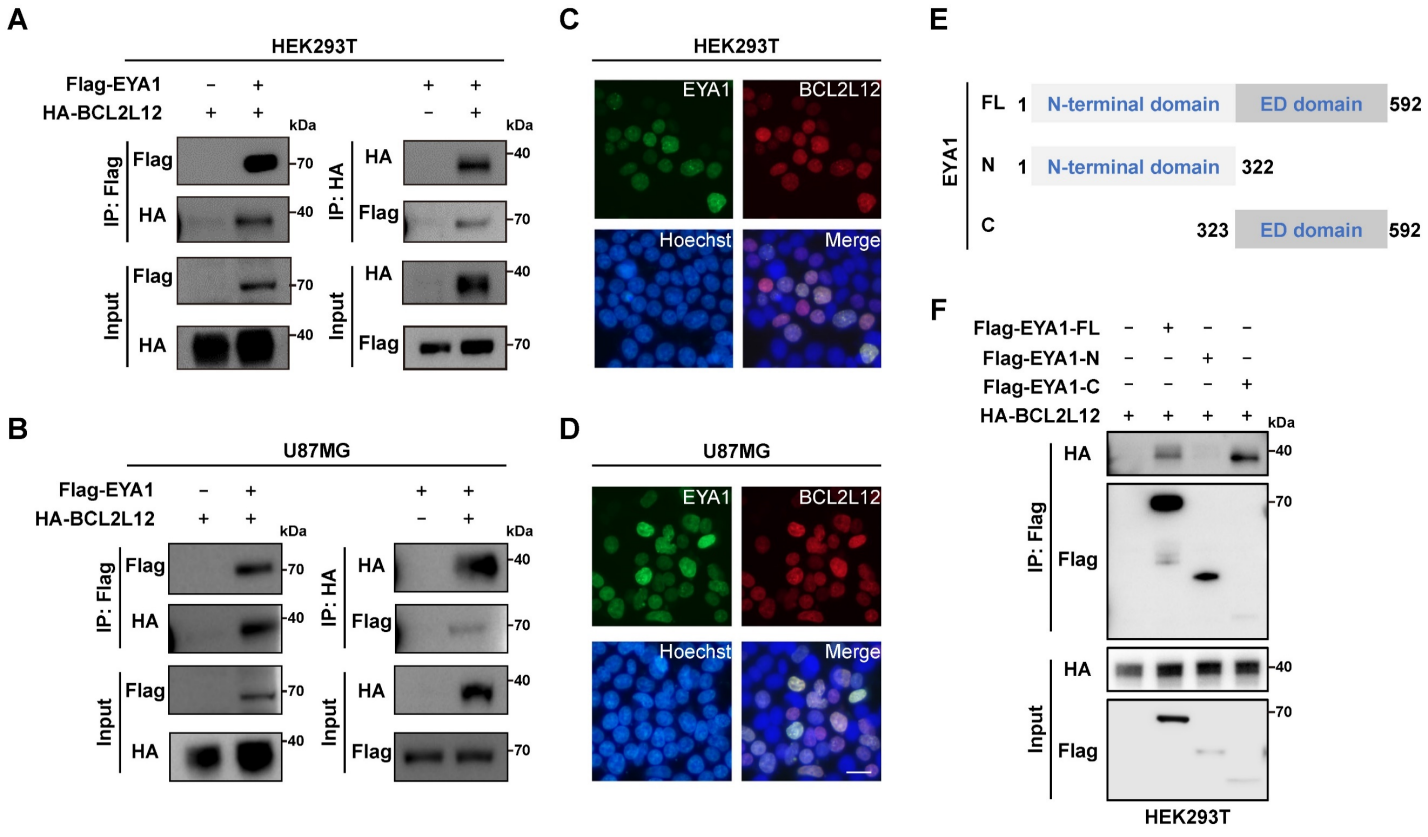
**EYA1 interacts and colocalizes with BCL2L12 in glioma cells.** A, Co-IP showing physical interaction between Flag-EYA1 and HA-BCL2L12 in HEK293T cells. B, Co-IP showing physical interaction between Flag-EYA1 and HA-BCL2L12 in U87MG cells. C, Live-cell imaging shows that EYA1 (green) colocalizes with BCL2L12 (red) in HEK293T cells. Bar=20 μm. D, Live-cell imaging shows that EYA1 (green) colocalizes with BCL2L12 (red) in U87MG cells. Bar= 20 μm. E, Schematic diagram of EYA1 truncations. F, Mapping essential domains mediating protein-protein interaction between EYA1 and BCL2L12.

Next, we investigated the biochemical interaction between EYA1 and BCL2L12 in mammalian cells and its potential impact on glioma development. Co-immunoprecipitation (Co-IP) experiments were conducted to verify the interaction between EYA1 and BCL2L12. HEK293T cells were transiently transfected with Flag-EYA1 or/and HA-BCL2L12, and cell lysates were immunoprecipitated with anti-Flag or anti-HA antibodies. The results indicated that Flag-EYA1 and HA-BCL2L12 pulled each other down, providing evidence of a biochemical interaction between the two proteins (Figure 2A). The same assay was performed using U87MG cells, and the results showed that EYA1 associated with BCL2L12 in U87MG cells (Figure 2B). To determine whether EYA1 and BCL2L12 colocalize in cells, GFP-tagged EYA1 and mCherry-tagged BCL2L12 were co-transfected into HEK293T and U87MG cells. The live-cell imaging results showed that EYA1 and BCL2L12 were primarily colocalized in the nucleus (Figure 2C-D).

EYA proteins have two conserved domains. Specifically, the N-terminal domain has both transcription activation and threonine phosphatase activities, while the C-terminal domain (also called EYA domain, ED domain) is highly conserved that has tyrosine phosphatase activity 22 (Figure S5D-E). To identify the EYA1 domain responsible for binding to BCL2L12, we created constructs for both the N-terminal domain (AA 1-322) and the C-terminal ED domain (AA 323-592) of EYA1 (Figure 2E). The Co-IP assay results revealed that EYA1 interacted with BCL2L12 through its C-terminal domain (Figure 2F). Taken together, these results demonstrate that EYA1 colocalizes and physically interacts with BCL2L12 in glioma cells.

### EYA1 regulates BCL2L12 turnover by dephosphorylating BCL2L12 at T33

**Figure 3:**
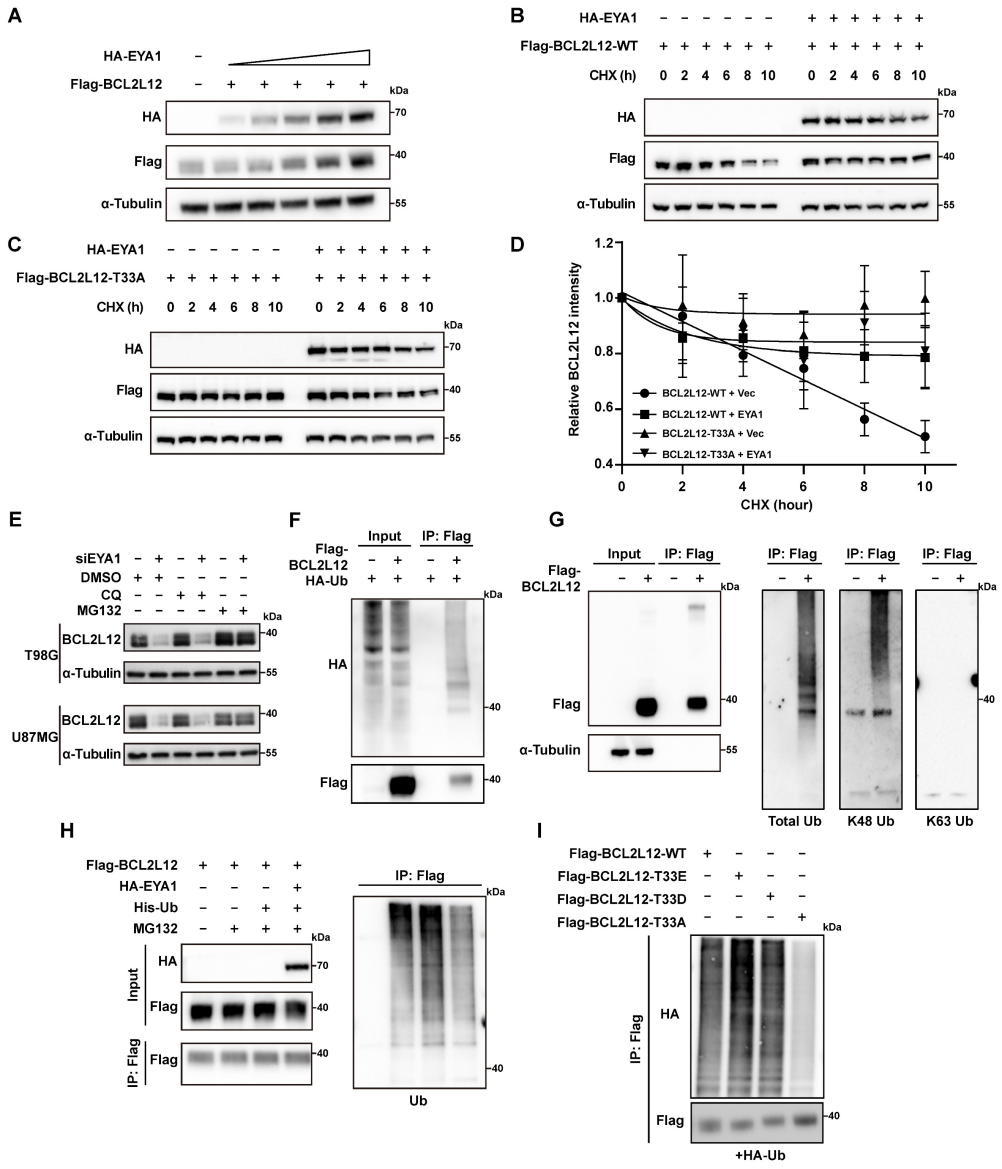
** EYA1 regulates BCL2L12 turnover.** A, Immunoblotting showing the level of BCL2L12 protein is elevated with EYA1 amount increasing. B-D, HEK293T cells were transfected with HA-EYA1 and Flag-tagged wild-type (B) or T33A (C) BCL2L12. 24 hours later, cells were treated with protein synthesis inhibitor cycloheximide as indicated. Protein level was detected, and protein stability curve was showed (D). E, Immunoblotting showing the level of BCL2L12 protein is restored by MG132 treatment in T98G and U87MG cells. F, BCL2L12 was modified by ubiquitination. HEK293T cells were transfected with HA-ubiquitin and BCL2L12 under MG132 treatment. Cells were lysed and subjected to IP using anti-flag beads and immunoblotting. G, IP-immunoblotting showing BCL2L12 was modulated by K48-polyubiquitination under MG132 treatment. H, EYA1 suppressed BCL2L12 ubiquitination. HEK293T cells were transfected with Flag-BCL2L12, HA-EYA1, His-Ub and treated with MG132 as indicated. Cells were lysed and subjected to Flag IP and immunoblotting using indicated antibodies. I, Ubiquitination assay showing the ubiquitination level of wild-type, phosphor-mimic (T33E and T33D), and phosphor-dead (T33A) BCL2L12 under MG132 treatment.

In previous studies, EYA1 regulates the substrate turnover by its threonine phosphatase activity. For instance, EYA1 dephosphorylates c-Myc at T58 to prevent it from E3 ligase recognition. This action increases c-Myc stability, promoting kidney development or mammary tumor progression 8, 26. Similarly, our earlier study indicates that EYA1 preserves Notch1 stability by dephosphorylating T2122, which is essential for normal craniofacial development 27. Given these findings, we examined whether EYA1 influences the turnover of BCL2L12. HEK293T cells were co-transfected with a constant amount of HA-BCL2L12 and increasing amounts of Flag-EYA1. The WB results indicated that increasing levels of EYA1 led to an increase in BCL2L12 protein levels, suggesting that EYA1 may enhance the stability of BCL2L12 (Figure 3A). To evaluate the dephosphorylation effects of EYA1 on BCL2L12 stability, HEK293T cells were transfected with wild-type (Flag-BCL2L12-WT) or phospho-dead BCL2L12 (Flag-BCL2L12-T33A) in the presence or absence of HA-EYA1, and the protein stability assay was employed using cycloheximide (CHX). Our results revealed that the wild-type BCL2L12 protein decreased to half its amount within 10 hours, while the overexpression of EYA1 significantly enhanced BCL2L12 protein stability (Figure 3B and D). Importantly, compared with wild-type BCL2L12, the phospho-dead BCL2L12 was much more stable, and the overexpression of EYA1 could not further increase T33A mutant's stability (Figure 3C-D). Together, these data indicate that the EYA1-mediated dephosphorylation of BCL2L12 at T33 plays a crucial role in regulating BCL2L12 stability.

To distinguish whether BCL2L12 is regulated by the proteasome or lysosome mediated degradation pathways, we conducted rescue experiments in EYA1 knockdown glioma cells using the proteasome inhibitor MG132 and the lysosome inhibitor chloroquine (CQ). The results indicated that MG132 significantly restored BCL2L12 levels after EYA1 suppression, while CQ had no effect. This suggests that BCL2L12 degradation occurs through the proteasome pathway (Figure 3E). This finding is consistent with previous studies that BCL2L12 undergoes proteasome degradation via the ubiquitination pathway 31, 32. To identify the ubiquitination sites in BCL2L12, we accessed the PhosphoSitePlus database 33. This database revealed three known ubiquitination sites in BCL2L12 (Figure S2D). We then generated a ubiquitination-deficient BCL2L12 mutant by substituting lysines K107, K122, and K143 with alanines. Ubiquitination assays showed that the mutant lacked ubiquitination compared to the wild-type BCL2L12. This finding confirms that the lysine residues K107, K122, and K143 are essential for ubiquitination in BCL2L12 (Figure S2E).

To examine the polyubiquitination type of BCL2L12, we first transfected HEK293T cells with Flag-BCL2L12 and HA-Ub, then immunoprecipitated the cell lysates using an anti-Flag antibody. Our immunoblotting analysis using anti-HA antibodies showed that BCL2L12 underwent ubiquitination (Figure 3F). To determine the polyubiquitination type on BCL2L12, the antibodies recognizing total ubiquitination, K48-linked polyubiquitination, and K63-linked polyubiquitination were used. The IP and immunoblotting assays revealed that BCL2L12 showed a ubiquitination signal with antibodies for total ubiquitination and K48-linked polyubiquitination, but not with K63-linked polyubiquitination (Figure 3G). This indicates that BCL2L12 is modified by K48-polyubiquitination, consistent with the understanding that K48-linked polyubiquitination primarily facilitates proteasomal recognition and degradation of substrates. Next, we aimed to determine if EYA1 regulates BCL2L12 stability by modulating its ubiquitination. We first compared the ubiquitination level of BCL2L12 with or without EYA1 overexpression. Indeed, our results indicated that the overexpression of EYA1 suppressed the total ubiquitination level of BCL2L12 (Figure 3H), which is consistent with our finding that EYA1 improves BCL2L12 stability (Figure 3A-D). Furthermore, to examine the effects of T33 dephosphorylation on BCL2L12 ubiquitination, we designed and constructed two mutants of BCL2L12 (T33E and T33D) that mimic the phosphorylated forms. Compared with the wild-type (WT) and phospho-mimic forms of BCL2L12 (T33E and T33D), the phospho-dead mutant (T33A) showed obviously lower level of ubiquitination of BCL2L12 (Figure 3I), which is in line with the higher stability of T33A mutant of BCL2L12 than that of wild-type BCL2L12 (Figure 3B-D). Taken together, these data demonstrate that EYA1 inhibits the ubiquitination and proteasomal degradation of BCL2L12 by dephosphorylating it at T33.

### EYA1-BCL2L12 signaling pathway is essential for glioma development

**Figure 4:**
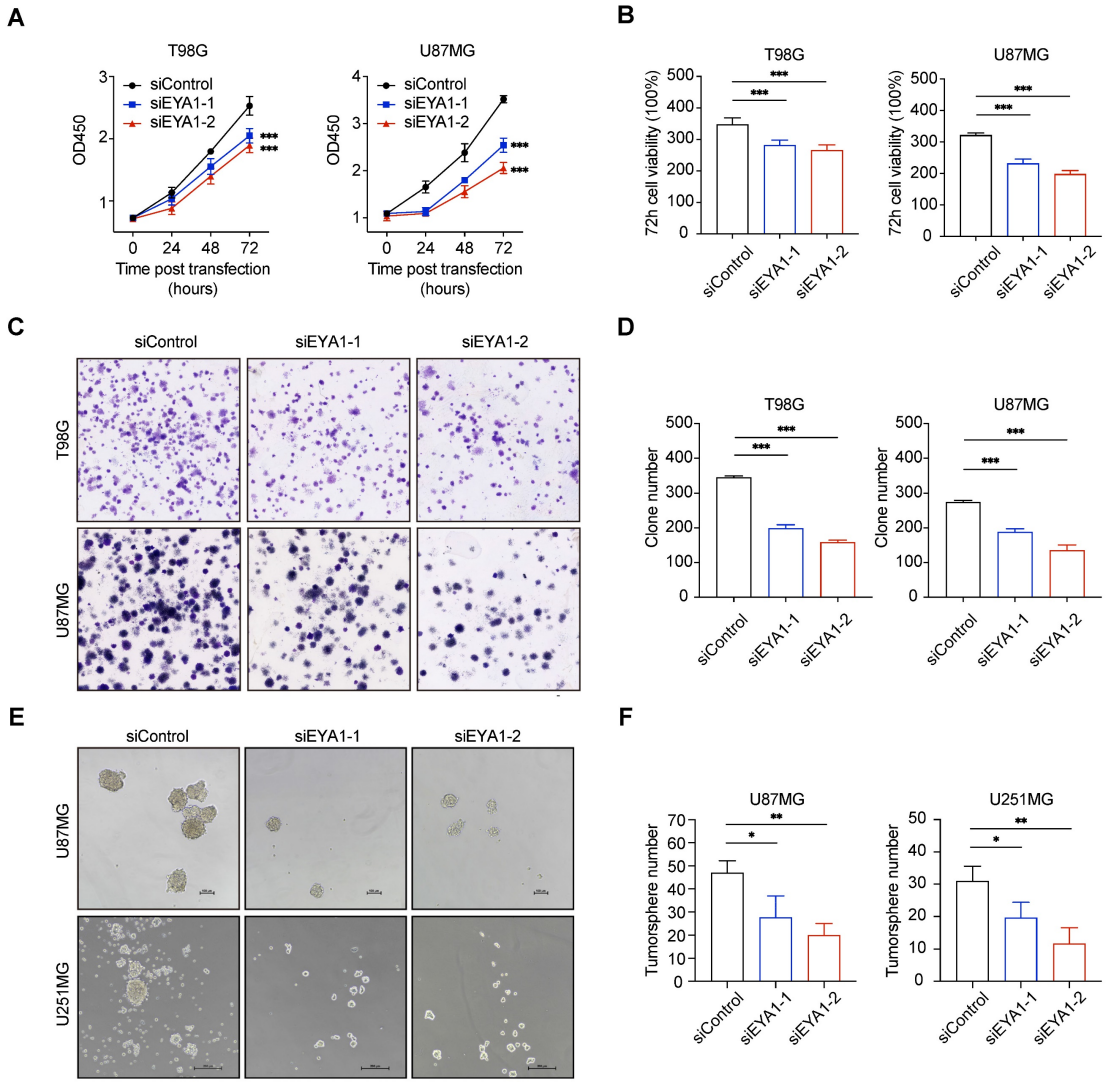
** EYA1 is essential for glioma cell growth *in vitro*.** A-B, T98G and U87MG cells transfected with siControl or siEYA1 (-1 and -2) were subjected to cell proliferation assay. Corresponding quantitative results are shown (B). C-D, T98G and U87MG cells transfected with siControl or siEYA1 (-1 and -2) were subjected to clone formation assay. Corresponding quantitative results are shown (D). E-F, U87MG and U251MG cells transfected with siControl or siEYA1 (-1 and -2) were subjected to tumorsphere formation assay. Corresponding quantitative results are shown (F). *P < 0.05, **P < 0.01, ***P < 0.001.

**Figure 5:**
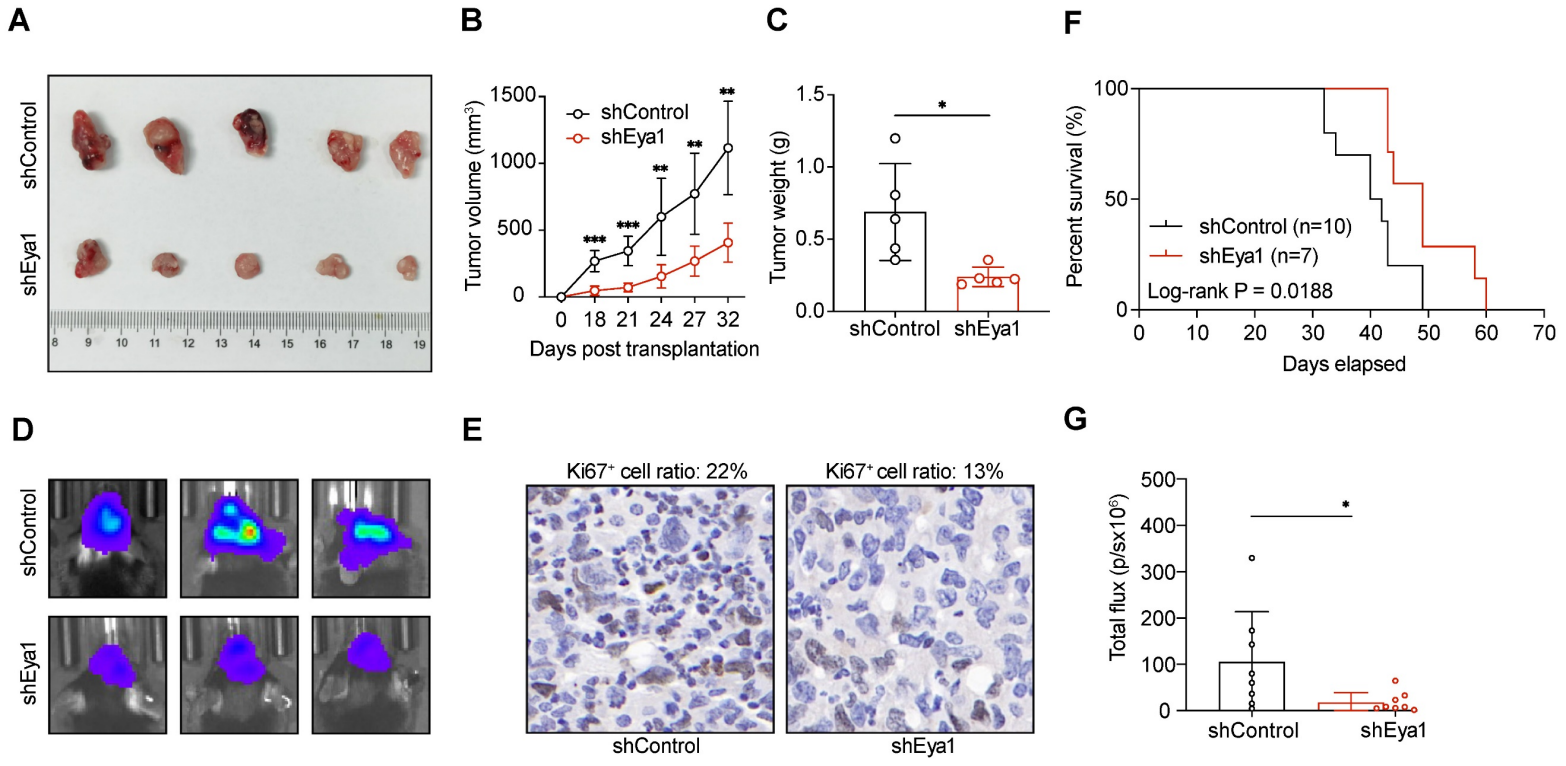
** EYA1 is essential for glioma tumor formation *in vivo*.** A-C, GL261 cells stably expressing shControl or shEya1 were subcutaneously injected into the right flanks of nude mice as indicated (n = 5 each group). After 32 days of injection, mice were sacrificed, and xenograft tumors were removed. Representative tumor images (A), tumor volume development curve (B), and tumor weight (C) are shown. D-G, GL261 cells stably expressing shControl or shEya1 were orthotopically transplanted into mice brain as indicated (n = 8 each group). Representative luminescence images (D), H.E. staining of mice brain tumor (E), survival curve (F), and statistical analysis of tumor luminescence (G) are shown. *P < 0.05, **P < 0.01, ***P < 0.001, ns, no significance.

To investigate the role of EYA1-BCL2L12 signaling pathway in glioma development, we initially knocked down endogenous EYA1 in the glioma cell lines T98G, U87MG, and U251MG using siRNAs. The expression status of EYA1 in the resultant cell lines was validated by immunoblotting (Figure S2C). In glioma cells, silencing EYA1 impaired cell proliferation (Figure 4A-B), clonogenicity ability (Figure 4C-D), and tumorsphere formation (Figure 4E-F). To assess the impact of EYA1 on glioma tumor formation *in vivo*, we used the luciferase-expressing mouse glioma cell line GL261 for tumor transplantation assays. Stable knockdown of mouse Eya1 in GL261 was performed and validated by immunoblot (Figure S2C). In the nude mouse model, Eya1 suppression significantly reduced tumor volume and weight compared to the control groups (Figure 5A-C). In the intracranial tumor transplantation mouse model, tumor formation was also inhibited by suppressing Eya1 (Figure 5D and G). The expression level of the cell proliferation marker, Ki67, was decreased in the tumors derived from Eya1-knockdown cells (Figure 5E). Moreover, silencing Eya1 increased the overall survival time of the mice (Figure 5F). Taken together, our data is consistent with the previous reports that EYA1 is essential for glioma cell growth *in vitro* and tumor formation *in vivo*.

**Figure 6:**
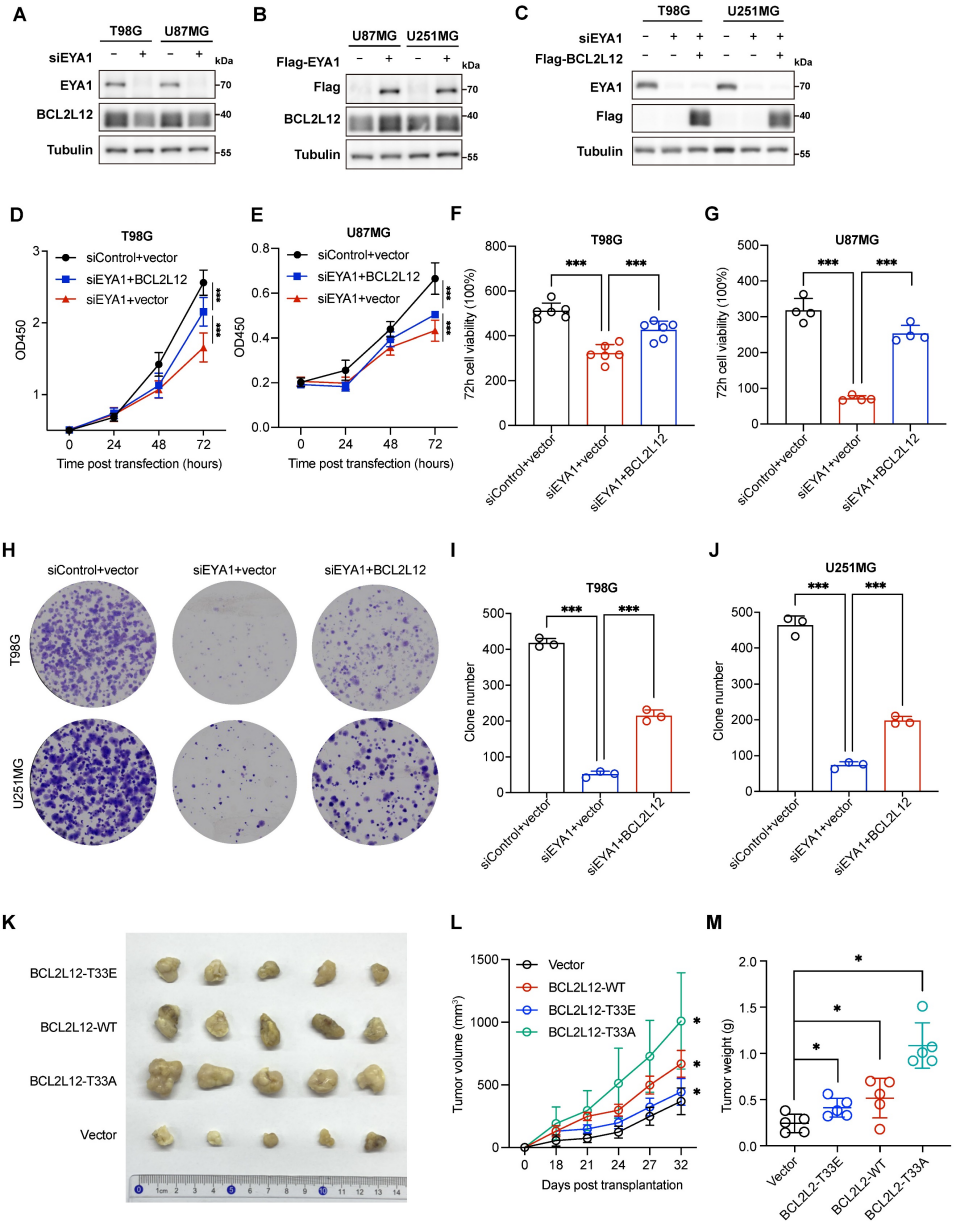
** EYA1-BCL2L12 signaling pathway is essential for glioma development.** A, T98G and U87MG cells transfected with siControl or siEYA1 were subjected to immunoblot analysis and BCL2L12 antibody was used to detect the endogenous BCL2L12 protein level. B, U87MG and U251MG cells overexpressing EYA1 were subjected to immunoblot analysis and BCL2L12 antibody was used to detect the endogenous BCL2L12 protein level. C, BCL2L12 is overexpressed in the EYA1-silencing cells in T98G and U87MG and cell lysates were subjected to immunoblot analysis for validation. D-G, T98G and U87MG cells transfected with indicated siRNAs or plasmids were subjected to CCK-8 assay (D and E). Corresponding quantitative results (F and G) are shown. H-J, T98G and U251MG cells transfected with indicated siRNAs or plasmids were subjected to clone formation assay (H). Corresponding quantitative results (I and J) are shown. K-M, U251MG cells stably expressing empty vector, wild-type, phosphor-mimic (T33E), and phosphor-dead (T33A) BCL2L12 were subcutaneously injected into the right flanks of nude mice as indicated (n = 5 each group). After 32 days of injection, mice were sacrificed, and xenograft tumors were removed. Representative tumor images (K), tumor volume development curve (L), and tumor weight (M) are shown. *P < 0.05, **P < 0.01, ***P < 0.001, ns, no significance.

We then asked whether BCL2L12 could mediate the oncogenic effects of EYA1 on glioma development. We first evaluated the effects of EYA1 on BCL2L12 expression in glioma cells. Suppressing EYA1 decreased BCL2L12 expression in T98G and U87MG cells, while overexpressing EYA1 raised BCL2L12 expression in U87MG and U251MG cells (Figure 6A-B). This finding supports our earlier observation that EYA1 regulates BCL2L12 turnover (Figure 3). To examine the role of BCL2L12 in EYA1's tumor-promoting effects, we overexpressed BCL2L12 in EYA1-knockdown cells (Figure 6C). Cellular assays revealed that overexpressing BCL2L12 partially restored cell proliferation in T98G and U87MG cells (Figure 6D-G). Similarly, the overexpression of BCL2L12 also partially rescued the cell clone formation ability in T98G and U251MG cells (Figure 6H-J). Collectively, these findings indicate that BCL2L12 partially mediates the oncogenic effects of EYA1 on glioma cell proliferation.

To investigate the effects of the phosphorylation status of T33 in BCL2L12 on glioma development, we stably overexpressed various versions of BCL2L12 in U251MG cells, including wild-type (WT), phospho-mimic (T33E), and phospho-dead (T33A) mutant, and xenografted them into the immunocompromised mice. Overexpression of BCL2L12-T33A significantly enhanced tumor formation, as indicated by increased tumor volume and weight, whereas cells overexpressing BCL2L12-T33E showed a weaker tumor formation ability, although still greater than that of the control group (Figure 6K-M). Taken together, these results suggest that EYA1-mediated dephosphorylation of BCL2L12 at T33 plays a crucial role in glioma tumor formation.

### EYA1 and BCL2L12 positively correlate in glioma patient samples

**Figure 7:**
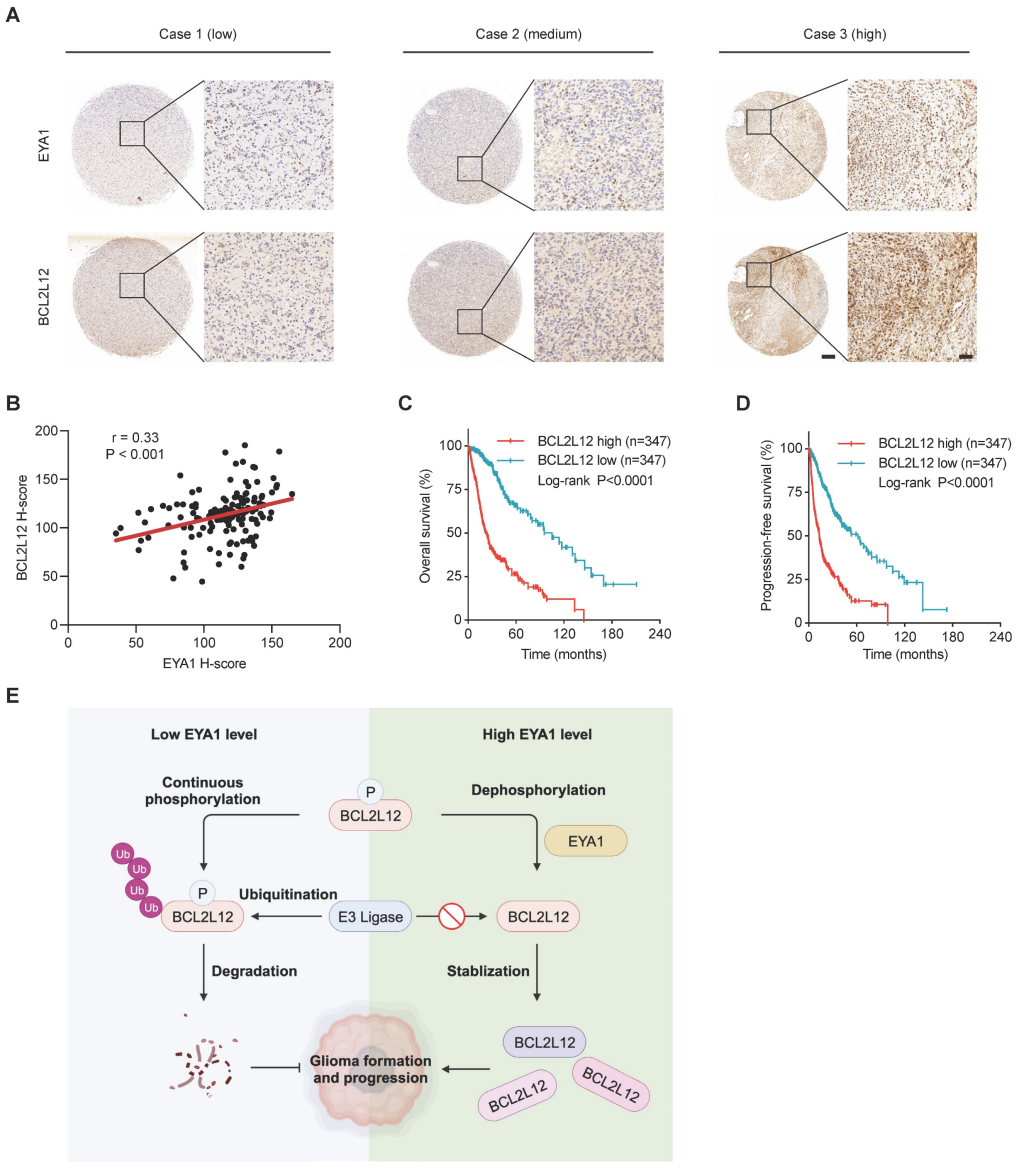
** EYA1 and BCL2L12 positively correlate in glioma patient samples.** A, Representative immunohistochemical staining of EYA1 and BCL2L12 proteins in glioma patient samples from low to high level. B, Pearson correlation analysis of EYA1 and BCL2L12 protein expression in glioma patient samples. C-D, Kaplan-Meier analysis of the overall survival (C) and progression-free survival (D) of glioma patients with high and low BCL2L12 expression from TCGA cohort. E, The proposed working model. This image was created with BioRender.com.

To evaluate the clinical relevance of EYA1 and BCL2L12 in glioma, we investigated their expression using immunohistochemical (IHC) staining on a glioma tissue microarray (TMA). In glioma patient samples, EYA1 was predominantly found in the nucleus, whereas BCL2L12 was mainly nuclear but also weakly detectable in the cytoplasm (Figure 7A). Pearson correlation analysis showed a positive correlation between EYA1 and BCL2L12 proteins in glioma patient samples (Figure 7B). Additionally, glioma patients with higher BCL2L12 mRNA levels experienced poorer overall and progression-free survival (Figure 7C-D and Figure S6). This suggests that BCL2L12 may serve as a valuable biomarker for predicting patient prognosis. Collectively, these findings indicate that the EYA1-BCL2L12 signaling pathway is important in clinical settings.

## Discussion

EYAs are unique protein phosphatases essential for the early embryonic development 34. Recent studies show that EYAs are upregulated in numerous brain tumors and are required for brain tumor formation and progression, including glioma 12-18. However, little is known about the specific targets that mediate the oncogenic roles of EYAs in brain tumor development. Using unbiased phosphoproteomics combined with a novel screening strategy, we identify BCL2L12 as an EYA1 threonine phosphatase substrate in glioma cells. BCL2L12 is an important oncoprotein that promotes cell proliferation and prevents apoptosis in glioma. We also describe a signaling pathway where EYA1 regulates BCL2L12 turnover by dephosphorylating T33, which contributes to glioma development (Figure 7E).

Like the well-known EYA1 substrates c-Myc and Notch1, BCL2L12 also features a conserved “TPXXSP” motif, where threonine serves as the dephosphorylation site for EYA1. This leads to the hypothesis that the “TPXXSP” motif is a common regulatory element for EYAs, suggesting that proteins with this motif may serve as potential substates for EYAs' threonine phosphatase activity. Our study offers new insights into the regulatory mechanisms of protein phosphorylation and dephosphorylation, making it interesting to validate this hypothesis in future research. In addition, atypical protein kinase C (aPKC), another substrate of EYA1 threonine phosphatase, does not contain the “TPXXSP” motif 35. EYA1 dephosphorylates aPKC at T410 to promote symmetric cell divisions during cerebellar development. Therefore, we still need to identify other potential substrates of EYA1 threonine phosphatase that do not follow the “TPXXSP” motif, as indicated by our phosphoproteomic results.

The crosstalk between different protein post-translational modification (PTM) is an important regulatory layer that affects protein structure and function. As two of the most abundant and best-studied PTMs, the interaction between phosphorylation and ubiquitination has especially been widely investigated 36-38. Interestingly, the degradation of two well-established EYA1 substrates, c-Myc and Notch1, is strictly regulated by its phosphorylation and dephosphorylation at certain sites 26, 27. The E3 ligase Fbw7 binds to c-Myc with phosphorylated T58, leading to c-Myc degradation via the proteasomal system 39. In embryonic kidney or breast cancer cells, EYA1 dephosphorylates T58 on c-Myc using its threonine phosphatase activity, thereby preventing its ubiquitination. The enhancement of c-Myc stability by EYA1 is essential for normal kidney development and breast cancer cell growth 8, 26. Similarly, our previous study also demonstrates that EYA1 dephosphorylates Notch1 at T2122 to prevent it from Fbw7-mediated ubiquitination-proteasomal degradation, which sustains Notch signaling activity in craniofacial development 27. BCL2L12 is reported to be degraded by the ubiquitination-proteasome system 31, 32. While it has been shown that EYA1 regulates substrates' turnover by its phosphatase activity, our study is the first report that the dephosphorylation of BCL2L12 by EYA1 prevents BCL2L12 from degradation. In our study, enforced EYA1 expression increases BCL2L12 expression in glioma cells, and, conversely, silencing EYA1 results in downregulation of BCL2L12. Moreover, the protein expression level of EYA1 and BCL2L12 is positively correlated in glioma patient samples, suggesting that this positive regulatory relevance between EYA1 and BCL2L12 is also important in clinical settings. However, the detailed mechanism by which EYA1 regulates BCL2L12 ubiquitination remains unclear. Specifically, the E3 ubiquitin ligase responsible for recognizing phosphorylated BCL2L12 degron centered at T33 remains to be characterized in the future investigations.

Sustaining proliferative signaling and evading apoptosis are two fundamental hallmarks of cancer 40. In previous study, EYA1 has been reported to be overexpressed in glioma tissues 12. EYA1 is crucial for maintaining the stemness and survival of GSCs. When EYA1 is knocked down, GSCs lose their ability to self-renew and undergo cell death 15. In comparison to normal brain tissues, BCL2L12 is also upregulated in glioma tumors 41, 42. Overexpression of BCL2L12 promotes the growth of glioma cells, resulting in poorer survival rates for mice transplanted with glioma cells that ectopically express BCL2L12 43, 44. Conversely, knocking down BCL2L12 inhibits glioma cell proliferation, induces apoptosis, and extends the survival time of mouse models 42, 45. The signaling transduction proposed in this study is consistent with the oncogenic roles of EYA1 and BCL2L12 in glioma development as previously reported. In line with previous reports, we also find that EYA1 inhibition suppresses glioma cell growth *in vitro* and *in vivo*. Our study importantly demonstrates that overexpression of BCL2L12 partially rescues the cell growth defects induced by silencing EYA1. This finding links the biological functions of EYA1 and BCL2L12, suggesting that BCL2L12 partially mediates the oncogenic effects of EYA1 in glioma. In addition, we demonstrate that the protein stability of the phospho-dead mutant of BCL2L12 (T33A) is much higher than that of wild-type BCL2L12, and that the phospho-dead mutant of BCL2L12 has stronger ability to promote tumor formation *in vivo* than that of wild-type or phospho-mimic mutant, suggesting the importance of the regulatory roles of EYA1 on BCL2L12 in glioma development.

Traditional glioma treatment, like radiotherapy and chemotherapy, primarily inhibit glioma development by inducing malignant cell death. However, glioma patients often experience therapeutic resistance. This resistance is due to the glioma cells' strong proliferation and anti-apoptotic abilities. BCL2L12 plays a crucial role in regulating glioma cell growth and survival. It is typically present at low levels in adult brain tissue but is elevated in glioma tumors, making it a rational therapeutic target. The RNAi-based spherical nucleic acids targeting BCL2L12 has been designed, developed, and applied in a phase 0 clinical study 45, 46. Currently, there are no small molecule drugs targeting BCL2L12 available in the clinic, partly due to the protein's flexible structure 46. Because BCL2L12 stability is controlled by ubiquitination-proteasome system, targeting BCL2L12 stabilizer makes it possible to regulate BCL2L12 activity alternatively. In this study, we demonstrate that EYA1 maintains BCL2L12 stability. Considering EYA1 is a phosphatase with a stable enzymatic pocket, targeting EYA1 phosphatase activity provides a promising strategy for resolving the undruggable problem of BCL2L12. Several EYAs' inhibitors have exhibited excellent anticancer effects in various cancers, such as lung cancer, breast cancer, medulloblastoma, and glioma 17, 18, 47-50. Therefore, the study presented here may be of clinical significance and provide a rationale for the development and application of EYAs' inhibitors for glioma treatment.

In summary, this study identifies BCL2L12 as a new substrate for EYA1 threonine phosphatase activity in glioma development. Disruption of the EYA1-BCL2L12 signaling pathway effectively inhibits glioma formation and progression, implicating EYA1 as a potential therapeutic target for glioma treatment.

## Methods

### Cell culture

The T98G, U87MG, U251MG, GL261, and HEK293T cells were obtained from ATCC. All cells were cultured DMEM (Gibco, CC11995500BT) supplemented with 10% FBS (Gibco, 10270106) and 1% Penicillin/Streptomycin (Gibco, 15140122) with 5% CO2 at 37 ◦C.

### RNAi and transfection

Target siRNAs and non-targeting controls (General biosystems) were transfected into the cells in combination with Lipofectamine RNAiMax Reagent (Invitrogen, 13778150) and Opti-MEM (Gibco, 31985-062) medium. Working concentration of all siRNAs was 20 nM. Knockdown efficiency was detected by immunoblotting. The targeting sequences of each siRNA were listed in Supplementary Table 1.

### Plasmid and transfection

The human EYA1 cDNA clone was purchased from YouBio (G124280). For overexpressing EYA1, the coding sequence of EYA1 was subcloned into the pcDNA3.1 and pLVX vectors. For overexpressing BCL2L12, the coding sequence of BCL2L12 was purchased from YouBio (G103652) and was subclone into the pcDNA3.1 and pLVX vectors. HA- Ubiquitin was used in ubiquitination assays (18712, Addgene). pLVX-BCL2L12-T33A, pLVX-BCL2L12-T87A, pLVX-BCL2L12-TTAA, pLVX-BCL2L12-T33E, and pLVX-BCL2L12-T33D were generated by site-directed mutagenesis and confirmed by DNA sequencing (primer sequences shown in Supplementary Table 1). The shRNA targeting Eya1 was generated in pLKO.1 according to standard protocol (shRNA oligos shown in Supplementary Table 1). Plasmids transfection was performed using Lipofectamine 3000 Reagent (Invitrogen, L3000015) according to the manufacturer's instructions.

### Lentivirus package and stable cell lines

For each virus package, 1 × 10^6^ HEK293 T cells were seeded into a 6 cm dish. After 24 h, 2 ug targeting plasmids mentioned above, 0.5 ug pMD2. G (Addgene, 12259), and 1.5 ug psPAX2 (Addgene, 12260) were mixed with Lipofectamine 3000 Reagent (Invitrogen, L3000015) and transfected into the HEK293 T cells. 48 h after transfection, the supernatant medium containing the lentivirus was collected and filtered by 0.45 µm syringe filter (Beyotime, FF365). The transduction was performed by incubating the viral containing medium to the targeting cells overnight in the presence of 8 ug/ml polybrene (OBiO, HYFW20190626003). 48 h after transduction, the cells were selected under 2.5 ug/ml puromycin (Gibco, A1113802) for further keeping and passaging.

### Western blotting

The immunoblotting experiments were performed as described previously 51. The antibodies used were listed in Supplementary table 3. The original uncropped western blots were uploaded.

### Tissue microarray analysis and immunohistochemistry staining

Human glioma tissue array was obtained from Alenabio (Bra1009b). The antibody against EYA1 (Proteintech, 22658-1-AP) and BCL2L12 (Proteintech, 16612-1-AP) were utilized for immunohistochemistry staining according to the manufacture's protocol. Image-Pro Plus (NIH) was used to determine the integral optical density (IOD) of the positively stained signal (brown) and area of haematoxylin-stained signal (blue) for each slide. The IOD/mm2 of the haematoxylin-stained area was then calculated and normalized as the relative staining score. Tissue microarrays were used according to Institutional Review Boards (IRB) protocols approved by Southern University of Science and Technology and Alenabio. All human subjects gave informed consent.

### Cell viability assay

The glioma cells (2000 cells per well) were seeded into a 96-well plate and cultured in 100 μl medium. When the cells attached to the well bottom,10 μl CCK-8 solution (YEASEN 40203ES92) was added and incubated at incubator for 2 hours. The absorbance was measured using a microplate reader (BioTek) at wavelength 450 nm. This value was regarded as the time point zero. Repeat to add the CCK-8 solution and measure values after 24, 48, and 72 hours. The cell viability was finally calculated.

### Clonogenicity assay

The glioma cells (500-1000 cells per well) were seeded into a 6-well plate and cultured in 2 ml medium. Around 10-14 days later, the single clones grew up until 50 cells aggregated into one single clone, the cells were fixed in 4% PFA and stained in 0.5% crystal violet (Solarbio, G1026). The clones were imaged and counted in Lionheart FX (BioTek).

### Tumorsphere formation assay

U87-MG and U251-MG (50-100 cells per well) were seeded into an ultra-low attachment 24-well plate (Corning 3473) and cultured in 500 μl tumorsphere medium. Tumorsphere medium consists of 500 ml DMEM/F12 (BI 01-172- 1ACS), 20 ng/ml EGF (Sino Biological 50482-MNCH), 10 ng/ml bFGF (Sino Biological 50037-M07E), 5 μg/ml insulin (Apexbio B7407), and 0.4% BSA (Sigma A4503). Around 10-14 days later, the tumorsphere could be observed under light microscope. The tumorsphere was imaged and counted under microscope (Nikon, ECLIPSE Ti2). The diameter of tumorsphere was measured and calculated using Image J.

### Subcutaneous tumorigenicity assay

BALB/c nude mice (4-6 weeks) obtained from the Animal Center in SUSTech were used for the subcutaneous tumorigenicity assay. 10 mice were randomly divided into two group. A total of 1×10^6^ GL261-luc cells (shcontrol or shEya1) resuspended in 100 µl PBS were injected to the mice' right back. Tumor length and width were measured every three days for one month. The tumor size was calculated as following formula: tumor volume (cm^3^) = (length × width^2^)/2. Finally, the animals were euthanized, and tumors were surgically dissected and weighed. The animal ethics was approved by the Animal Experimentation Ethics Committee in SUSTech, and all mice were maintained in the Laboratory Animal Center in SUSTech.

### Intracranial tumorigenicity assay

C57BL/6J mice aged 6-8 weeks were anesthetized in the anesthesia system (isoflurane 2L/min, oxygen 1L/min). Fixed the animal in a stereotaxic, applied eye ointment to protect animals' eyes from light damage. Then sterilized and dehaired the scalp. A sagittal incision over the parieto-occipital bone was achieved using a sterile scalpel, approximately 1 cm long. To emerge bregma, 3% hydrogen peroxide solution was smeared on the skull surface. Used a microdrill to puncture the skull at 2 mm to the right of the bregma and 2 mm anterior to the coronal suture to provide an opening for the injection of tumor cells. Loaded the syringe with 2×10^5^ GL261-luc cells resuspended in 5 µl PBS, placed the syringe to mice brain onto 3 mm depth through the hole created before and retracted back 1 mm, and slowly injected the cells at 1 µl/min. After injection completion, leaved the syringe for another minute to avoid spill, then slowly withdrawal. Finally, sterilized and sewed up the incision. After one week, the bioluminescence intensity of intracranial tumor could be measured using luciferin (15mg/ml) in IVIS Spectrum (PerkinElmer). The animal ethics was approved by the Animal Experimentation Ethics Committee in SUSTech, and all mice were maintained in the Laboratory Animal Center in SUSTech.

### Phosphoproteome

U87MG cell lines stably expressing Flag-tagged EYA1 or empty vector were generated as described as above. A total of 2×10^7^ cells for each group were collected and delivered to Novogene after quick freezing in liquid nitrogen. The protein extraction, protein digestion, phosphorus peptides enrichment, mass spectrometry analysis, and data analysis were performed under standard protocol in Novogene (described in supplementary information).

### IP and Co-IP

The plasmids encoding Flag-tagged protein were transiently transfected into HEK293T cells in a 10 cm dish. 48 hours later, the cells were collected and lysed in NP-40 buffer at 4℃ for 30 minutes with gentle rotation. The cell lysis was centrifuged at 12000 rpm at 4℃ for 10 minutes. The supernatant was incubated with anti-Flag M2 affinity gel (Sigma A2220) at 4℃ for 2-4 hours with gentle rotation. Washed the gel three times by cold NP-40 buffer. The IP products in beads could be used for further protein digestion and proteomics analysis, denatured for Coomassie blue staining, immunoblotting analysis, and biochemical activity assay.

### Phosphatase assay

HA-EYA1 or Flag-BCL2L12 and various mutations were transiently transfected into a 10-cm dish HEK293T cells as indicated. 48 hours later, Flag-tagged BCL2L12 was enriched by immunoprecipitation as described above. The IP products were denatured for immunoblotting analysis. The phosphorous level of Threonine-Proline motif was measured using P-Thr-Pro-101 antibody (CST 9391). The total basal level of immunoprecipitated BCL2L12 was measured by Flag-HRP antibody (Sigma A8592). The quantification of phosphorylation level of Threonine-Proline was determined using the ratio of the intensity of P-Thr-Pro and Flag.

### Protein stability assay

Transfected HEK293T cells were seeded into a 12-well dish. 48 hours later, the culture medium was replaced by fresh medium containing 200 µM cycloheximide (MCE HY-12320) to stop new protein synthesis. Cells were collected and lysed every two hours at indicated time point for immunoblotting. The band intensity was determined by Image J.

### Ubiquitination assay

Flag-tagged BCL2L12 and certain plasmids were transfected into HEK293T cells in 10cm dish. After 36 hours, culture medium was replaced by fresh culture medium containing 20 µM MG132 (Selleck S2619) to suppress proteosome-mediated protein degradation. Transfected HEK293T cells were collected and lysed in NP-40 buffer as described above. The IP products were finally denatured and prepared for immunoblotting analysis using the antibody against ubiquitin.

### Public database mining

The expression matrix of scRNA-seq data of EYA1 in different human tumors was generated in TISCH (http://tisch.comp-genomics.org/home/). The transcriptome data of LGG and GBM from TCGA were obtained from UCSC Xena (https://xenabrowser.net/) and CGGA (http://www.cgga.org.cn/). Kaplan-Meier estimates and log-rank test were used to develop survival curves and survival analysis.

### Statistical analysis

All experimental data was represented as mean ± SD. The significance between two groups was determined by two-pair t test. Significance was considered as P value less than 0.05 (* P < 0.05, ** P < 0.01, and * P < 0.001). The n numbers for each group and group numbers are indicated in the figure or figure legends. GraphPad Prism 9 was used to perform statistical analysis.

## Supplementary Material

Supplementary methods, figures and tables.

Supplementary data.

## Abbreviations

BCL2L12: BCL2-like 12
T33: threonine 33
EYA: eyes absent
GSCs: glioblastoma stem cells
Y142: tyrosine 142
Y445: tyrosine 445
T58: threonine 58
T2122: threonine 2122
TISCH: Tumor Immune Single Cell Hub
CCLE: Cancer Cell Line Encyclopedia
P34: proline 34
Co-IP: Co-immunoprecipitation
CQ: chloroquine
K107: lysine 107
WT: wildtype
IHC: immunohistochemistry

## References

[1] Sung H, Ferlay J, Siegel RL, Laversanne M, Soerjomataram I, Jemal A (2021). Global Cancer Statistics 2020: GLOBOCAN Estimates of Incidence and Mortality Worldwide for 36 Cancers in 185 Countries. CA Cancer J Clin.

[2] Tan AC, Ashley DM, Lopez GY, Malinzak M, Friedman HS, Khasraw M (2020). Management of glioblastoma: State of the art and future directions. CA Cancer J Clin.

[3] Gatto L, Franceschi E, Tosoni A, Di Nunno V, Bartolini S, Brandes AA (2023). Glioblastoma treatment slowly moves toward change: novel druggable targets and translational horizons in 2022. Expert Opin Drug Discov.

[4] Castro MG, Cowen R, Williamson IK, David A, Jimenez-Dalmaroni MJ, Yuan X (2003). Current and future strategies for the treatment of malignant brain tumors. Pharmacol Ther.

[5] Xu J, Li J, Zhang T, Jiang H, Ramakrishnan A, Fritzsch B (2021). Chromatin remodelers and lineage-specific factors interact to target enhancers to establish proneurosensory fate within otic ectoderm. Proc Natl Acad Sci U S A.

[6] Zou D, Erickson C, Kim EH, Jin D, Fritzsch B, Xu PX (2008). Eya1 gene dosage critically affects the development of sensory epithelia in the mammalian inner ear. Hum Mol Genet.

[7] Xu PX, Adams J, Peters H, Brown MC, Heaney S, Maas R (1999). Eya1-deficient mice lack ears and kidneys and show abnormal apoptosis of organ primordia. Nat Genet.

[8] Xu J, Wong EY, Cheng C, Li J, Sharkar MT, Xu CY (2014). Eya1 interacts with Six2 and Myc to regulate expansion of the nephron progenitor pool during nephrogenesis. Dev Cell.

[9] Xu PX, Zheng W, Laclef C, Maire P, Maas RL, Peters H (2002). Eya1 is required for the morphogenesis of mammalian thymus, parathyroid and thyroid. Development.

[10] Zou D, Silvius D, Davenport J, Grifone R, Maire P, Xu PX (2006). Patterning of the third pharyngeal pouch into thymus/parathyroid by Six and Eya1. Dev Biol.

[11] Laclef C, Souil E, Demignon J, Maire P (2003). Thymus, kidney and craniofacial abnormalities in Six 1 deficient mice. Mech Dev.

[12] Auvergne RM, Sim FJ, Wang S, Chandler-Militello D, Burch J, Al Fanek Y (2013). Transcriptional differences between normal and glioma-derived glial progenitor cells identify a core set of dysregulated genes. Cell Rep.

[13] Zhang K, Zhao H, Zhang K, Hua C, Qin X, Xu S (2020). Chromatin-regulating genes are associated with postoperative prognosis and isocitrate dehydrogenase mutation in astrocytoma. Ann Transl Med.

[14] Li Z, Qiu R, Qiu X, Tian T (2018). EYA4 Promotes Cell Proliferation Through Downregulation of p27Kip1 in Glioma. Cell Physiol Biochem.

[15] Kim J, She C, Potez M, Huang P, Wu Q, Prager BC (2021). Phage display targeting identifies EYA1 as a regulator of glioblastoma stem cell maintenance and proliferation. Stem Cells.

[16] Wen Z, Liang C, Pan Q, Wang Y (2017). Eya2 overexpression promotes the invasion of human astrocytoma through the regulation of ERK/MMP9 signaling. Int J Mol Med.

[17] Zhang G, Dong Z, Gimple RC, Wolin A, Wu Q, Qiu Z (2021). Targeting EYA2 tyrosine phosphatase activity in glioblastoma stem cells induces mitotic catastrophe. J Exp Med.

[18] Wolin AR, Vincent MY, Hotz T, Purdy SC, Rosenbaum SR, Hughes CJ (2023). EYA2 Tyrosine Phosphatase Inhibition Reduces MYC and Prevents Medulloblastoma Progression. Neuro Oncol.

[19] Li X, Oghi KA, Zhang J, Krones A, Bush KT, Glass CK (2003). Eya protein phosphatase activity regulates Six1-Dach-Eya transcriptional effects in mammalian organogenesis. Nature.

[20] Okabe Y, Sano T, Nagata S (2009). Regulation of the innate immune response by threonine-phosphatase of Eyes absent. Nature.

[21] Tadjuidje E, Hegde RS (2013). The Eyes Absent proteins in development and disease. Cell Mol Life Sci.

[22] Hughes CJ, Alderman C, Wolin AR, Fields KM, Zhao R, Ford HL (2024). All eyes on Eya: A unique transcriptional co-activator and phosphatase in cancer. Biochim Biophys Acta Rev Cancer.

[23] Cook PJ, Ju BG, Telese F, Wang X, Glass CK, Rosenfeld MG (2009). Tyrosine dephosphorylation of H2AX modulates apoptosis and survival decisions. Nature.

[24] Krishnan N, Jeong DG, Jung SK, Ryu SE, Xiao A, Allis CD (2009). Dephosphorylation of the C-terminal tyrosyl residue of the DNA damage-related histone H2A.X is mediated by the protein phosphatase eyes absent. J Biol Chem.

[25] Nelson CB, Rogers S, Roychoudhury K, Tan YS, Atkinson CJ, Sobinoff AP (2024). The Eyes Absent family members EYA4 and EYA1 promote PLK1 activation and successful mitosis through tyrosine dephosphorylation. Nat Commun.

[26] Li J, Rodriguez Y, Cheng C, Zeng L, Wong EYM, Xu CY (2017). EYA1's Conformation Specificity in Dephosphorylating Phosphothreonine in Myc and Its Activity on Myc Stabilization in Breast Cancer. Mol Cell Biol.

[27] Zhang H, Wang L, Wong EYM, Tsang SL, Xu PX, Lendahl U (2017). An Eya1-Notch axis specifies bipotential epibranchial differentiation in mammalian craniofacial morphogenesis. Elife.

[28] Wang L, Xie J, Zhang H, Tsang LH, Tsang SL, Braune EB (2020). Notch signalling regulates epibranchial placode patterning and segregation. Development.

[29] Sun D, Wang J, Han Y, Dong X, Ge J, Zheng R (2021). TISCH: a comprehensive web resource enabling interactive single-cell transcriptome visualization of tumor microenvironment. Nucleic Acids Res.

[30] Ghandi M, Huang FW, Jane-Valbuena J, Kryukov GV, Lo CC, McDonald ER 3rd (2019). Next-generation characterization of the Cancer Cell Line Encyclopedia. Nature.

[31] Hornbeck PV, Chabra I, Kornhauser JM, Skrzypek E, Zhang B (2004). PhosphoSite: A bioinformatics resource dedicated to physiological protein phosphorylation. Proteomics.

[32] Yang J, Hong Y, Wang W, Wu W, Chi Y, Zong H (2009). HSP70 protects BCL2L12 and BCL2L12A from N-terminal ubiquitination-mediated proteasomal degradation. FEBS Lett.

[33] Hornbeck PV, Zhang B, Murray B, Kornhauser JM, Latham V, Skrzypek E (2015). PhosphoSitePlus, 2014: mutations, PTMs and recalibrations. Nucleic Acids Res.

[34] Vartuli RL, Zhou H, Zhang L, Powers RK, Klarquist J, Rudra P (2018). Eya3 promotes breast tumor-associated immune suppression via threonine phosphatase-mediated PD-L1 upregulation. J Clin Invest.

[35] Merk DJ, Zhou P, Cohen SM, Pazyra-Murphy MF, Hwang GH, Rehm KJ (2020). The Eya1 Phosphatase Mediates Shh-Driven Symmetric Cell Division of Cerebellar Granule Cell Precursors. Dev Neurosci.

[36] Filipcik P, Curry JR, Mace PD (2017). When Worlds Collide-Mechanisms at the Interface between Phosphorylation and Ubiquitination. J Mol Biol.

[37] Swaney DL, Beltrao P, Starita L, Guo A, Rush J, Fields S (2013). Global analysis of phosphorylation and ubiquitylation cross-talk in protein degradation. Nat Methods.

[38] Barbour H, Nkwe NS, Estavoyer B, Messmer C, Gushul-Leclaire M, Villot R (2023). An inventory of crosstalk between ubiquitination and other post-translational modifications in orchestrating cellular processes. iScience.

[39] Yada M, Hatakeyama S, Kamura T, Nishiyama M, Tsunematsu R, Imaki H (2004). Phosphorylation-dependent degradation of c-Myc is mediated by the F-box protein Fbw7. EMBO J.

[40] Hanahan D (2022). Hallmarks of Cancer: New Dimensions. Cancer Discov.

[41] Chou CH, Chou AK, Lin CC, Chen WJ, Wei CC, Yang MC (2012). GSK3beta regulates Bcl2L12 and Bcl2L12A anti-apoptosis signaling in glioblastoma and is inhibited by LiCl. Cell Cycle.

[42] Stegh AH, Kim H, Bachoo RM, Forloney KL, Zhang J, Schulze H (2007). Bcl2L12 inhibits post-mitochondrial apoptosis signaling in glioblastoma. Genes Dev.

[43] Stegh AH, Brennan C, Mahoney JA, Forloney KL, Jenq HT, Luciano JP (2010). Glioma oncoprotein Bcl2L12 inhibits the p53 tumor suppressor. Genes Dev.

[44] Stegh AH, Kesari S, Mahoney JE, Jenq HT, Forloney KL, Protopopov A (2008). Bcl2L12-mediated inhibition of effector caspase-3 and caspase-7 via distinct mechanisms in glioblastoma. Proc Natl Acad Sci U S A.

[45] Jensen SA, Day ES, Ko CH, Hurley LA, Luciano JP, Kouri FM (2013). Spherical nucleic acid nanoparticle conjugates as an RNAi-based therapy for glioblastoma. Sci Transl Med.

[46] Kumthekar P, Ko CH, Paunesku T, Dixit K, Sonabend AM, Bloch O (2021). A first-in-human phase 0 clinical study of RNA interference-based spherical nucleic acids in patients with recurrent glioblastoma. Sci Transl Med.

[47] Zhou H, Blevins MA, Hsu JY, Kong D, Galbraith MD, Goodspeed A (2020). Identification of a Small-Molecule Inhibitor That Disrupts the SIX1/EYA2 Complex, EMT, and Metastasis. Cancer Res.

[48] Anantharajan J, Zhou H, Zhang L, Hotz T, Vincent MY, Blevins MA (2019). Structural and Functional Analyses of an Allosteric EYA2 Phosphatase Inhibitor That Has On-Target Effects in Human Lung Cancer Cells. Mol Cancer Ther.

[49] Wang Y, Pandey RN, Riffle S, Chintala H, Wikenheiser-Brokamp KA, Hegde RS (2018). The Protein Tyrosine Phosphatase Activity of Eyes Absent Contributes to Tumor Angiogenesis and Tumor Growth. Mol Cancer Ther.

[50] Hwang GH, Pazyra-Murphy MF, Seo HS, Dhe-Paganon S, Stopka SA, DiPiazza M (2024). A Benzarone Derivative Inhibits EYA to Suppress Tumor Growth in SHH Medulloblastoma. Cancer Res.

[51] Wei T, Lin R, Fu X, Lu Y, Zhang W, Li Z (2022). Epigenetic regulation of the DNMT1/MT1G/KLF4/CA9 axis synergises the anticancer effects of sorafenib in hepatocellular carcinoma. Pharmacol Res.

[52] Ma J, Chen T, Wu S, Yang C, Bai M, Shu K (2019). iProX: an integrated proteome resource. Nucleic Acids Res.

[53] Chen T, Ma J, Liu Y, Chen Z, Xiao N, Lu Y (2022). iProX in 2021: connecting proteomics data sharing with big data. Nucleic Acids Res.

