# Supplementary material for: Protein phosphatase EYA1 regulates the dephosphorylation and turnover of BCL2L12 to promote glioma development
Source: Int J Biol Sci. 2025 Jan 13;21(3):1081–96. doi: 10.7150/ijbs.99619 (PMC11781168; doi:10.7150/ijbs.99619)
Supplement: Supplementary file 1 — Supplementary methods, figures and tables. [file ijbsv21p1081s1.pdf]

Supplementary Information

Supplementary methods

Phosphoproteomic analysis: The tissue samples were taken from the refrigerator at -80°C, ground into powder at low temperature, and quickly transferred to the liquid nitrogen pre-cooled centrifugal tube. Appropriate amount of protein lysis solution (100 mM ammonium bicarbonate, 6 M urea, 0.2% SDS, pH=8) was added, and mixed by shaking. Centrifugation was performed at 4°C at 12000 g for 15 min, and the supernatant was added with a final concentration of 10 mM DTTred for reaction at 56°C for 1 h. After that, a sufficient amount of IAM was added, and the reaction was performed at room temperature under dark conditions for 1 h. 4 times volume of -20°C precooled acetone was added for precipitation at -20°C for at least 2 h and centrifuged at 12000 g at 4°C for 15 min to collect the precipitation. After that, 1mL-20 °C pre-cooled acetone was added for resuspended, and the precipitate was cleaned. The precipitate was collected and dried by centrifugation at 12000 g at 4°C for 15 min. An appropriate amount of protein solution (6 M urea, 100 mM TEAB, pH=8.5) was added to dissolve the protein precipitate.

Using Bradford Protein quantification Kit, prepare a BSA standard protein solution according to the instructions with a concentration gradient of 0-0.5μg/μL. BSA standard protein solutions with different concentration gradients and sample solutions with different dilution ratios were added to the 96-well plate, and the volume was supplemented to 20 μL, and each gradient was repeated 3 times. 180 μL G250 staining solution was quickly added and placed at room temperature for 5 min. The absorbance at 595 nm was measured. The absorbance of standard protein solution was used to draw the standard curve and calculate the protein concentration of the sample to be tested. 20 μg protein samples were taken for 12% SDS-PAGE gel electrophoresis. The gel electrophoresis conditions were 80 V and 20 min for concentrated gel electrophoresis, and 120 V and 90 min for separated gel electrophoresis. After electrophoresis, Coomassie bright blue R-250 staining was performed until the bands were clear.

5 mg protein samples were taken from each sample, and protein solution was added to fill the volume to 2 mL. 50 μL 1 μg/μL trypsin and 10 mL 50 mM TEAB buffer were added. After mixing, enzyme digestion was performed overnight at 37°C. Add an equal volume of 1% formic acid, mix well, centrifuge at 12000 g at room temperature for 5 min, take the supernatant and slowly pass it through the C18 de-salting column, then use cleaning solution (0.1% formic acid, 4% acetonitrile) for continuous cleaning for 3 times. Add appropriate amount of eluent (0.1% formic acid, 75% acetonitrile) for two consecutive elution and freeze dry.

Add an appropriate amount of iMAC-Fe column cleaning solution (250 mM acetic acid, 30% acetonitrile) to dissolve the freeze-dried powder (pH=2.5-3.0), centrifuge it at 12000 g at 4°C for 5 min, take the supernatant and add it to the iMAC-Fe column treated with cleaning solution, and incubate it at room temperature for 25-30min. After centrifugation at 2000 g for 30 s, add cleaning solution and water for cleaning once. Centrifuge at 2000 g for 30 s, discard the liquid receiving tube, replace it with a new and clean centrifugal tube, add 400 mM ammonia water for elution, and freeze dry the eluent.

Supplementary tables

Supplementary table 1. DNA constructs

**Name**	**Backbone**	**Source**
pLVX-3Flag vector	pLVX-Hyg	Prof. Chuanyue Wu
pLVX-3Flag-EYA1	pLVX-Hyg	This paper
pLVX-3Flag-Eya1	pLVX-Hyg	This paper
3HA-EYA1	pcDNA3.1	GeneCopoeia
pcDNA3.1-3HA vector	pcDNA3.1	Prof. Xingzhi Xu
pLVX-GFP-EYA1	pLVX-AcGFP1-N1	YouBio
pLVX-mcherry-SIX1	pLVX-mCherry-N1	YouBio
pcDNA3.1-3HA-BCL2L12	pcDNA3.1-3HA vector	YouBio
pLVX-mcherry-BCL2L12	pLVX-mCherry-N1	This paper
pLVX-3Flag-BCL2L12	pLVX-Hyg	This paper
pLVX-3Flag-T33A	pLVX-Hyg	This paper
pLVX-3Flag-T87A	pLVX-Hyg	This paper
pLVX-3Flag-TAA	pLVX-Hyg	This paper
His-ubiquitin	pcDNA	Addgene
shRNA-NC	pLKO.1	This paper
shEya1	pLKO.1	This paper

Supplementary table 2. Oligo sequences for DNA construction and RNA interfering

<table>
<tr><th>Primer</th><th>Sequence</th><th>Application</th></tr>
<tr><td>pLVX-3Flag-EYA1-F</td><td>ATGACGATGACAAGACTAGTATGGAAATG
CAGGATCTAACCAGCC</td><td rowspan="3">Molecular cloning</td></tr>
<tr><td>pLVX-3Flag-EYA1-R</td><td>AGGGGCGGGATCCGCGGCCGCTTACAGG
TACTCCAGTTCCAAGGCA</td></tr>
<tr><td>pLVX-3Flag-Eya1-F</td><td>GGACTAGTATGGAAATGCAGGATCTAACC
AGCCCG</td></tr>
</table>

pLVX-3Flag-Eya1-R	ATAAGAATGCGGCCGCTTACAGGTACTCT AATTCCAAG	
pEGFP-EYA1-F	CCGCTCGAGCAGAAATGCAGGATCTAACC	
pEGFP-EYA1-R	CCGGAATTCCTACAGGTACTCCAGTTCC	
pLVX-3Flag- BCL2L12-F	ATGACGATGACAAGACTAGTATGGCAGGC TCTGAAGAGCTGGGGC	
pLVX-3Flag- BCL2L12-R	AGGGGCGGGATCCGCGGCCGCTCAGTCC AATGGCAAGTTCAAGTCC	
pLVX-mcherry-BCL2L12-F	GGACTCAGATCTCGAGACTACAAAGACCA TGACGGTGATTATAAAGA	
pLVX-mcherry-BCL2L12-R	TAGATCCGGTGGATCTCAGTCCAATGGCA AGTTCAAGTCCACG	
T33A-F	CTGTTCCAGCGCCACCTAGAAGCCCTGCC	Site mutagenesis
T33A-R	TAGGTGGCGCTGGAACAGGAGACCCGGC	
T87A-F	CCCAGCTGCGCCAGACTTCTATGCTTTGG TGGC	
T87A-R	GTCTGGCGCAGCTGGGCCAGGCTCTAAA C	
shEya1 Forward	CCGGGCAGGACTATCCGTCTTATCTCGAG ATAAGACGGATAGTCCTGCTTTTTG	shRNA construction
shEya1 Reverse	AATTCAAAAGCAGGACTATCCGTCTTATCT CGAGATAAGACGGATAGTCCTGC	

Supplementary table 3. Antibodies

Antibody	Host	Vendor	Catalog number	Application
EYA1	Rabbit	Proteintech	22658-1-AP	WB 1:1000<br>IHC 1:500
EYA1	Rabbit	Invitrogen	703582	WB1:1000

Flag	Mouse	Sigma	F1804	WB 1:2000<br>IP 1:1000
DYKDDDK	Rabbit	CST	14793	WB 1:1000<br>IP 1:1000
Flag-HRP	Mouse	Sigma	A8592	WB 1:2000
HA	Rabbit	CST	3724	WB 1:2000<br>IP 1:1000
HA-HRP	Rabbit	CST	14031	WB 1:2000
P-Thr-Pro-101	Mouse	CST	9391	WB 1:1000
Ubiquitin	Rabbit	CST	3933	WB 1:1000
GAPDH-HRP	Mouse	Proteintech	HRP-60004	WB 1:5000
β-actin-HRP	Mouse	Proteintech	HRP-60008	WB 1:5000
α-tubulin-HRP	Mouse	Proteintech	HRP-66031	WB 1:5000
Anti-rabbit IgG, HRP-linked Antibody	Goat	CST	7074	WB 1:5000
Anti-mouse IgG, HRP-linked Antibody	Horse	CST	7076	WB 1:5000

Supplementary figures

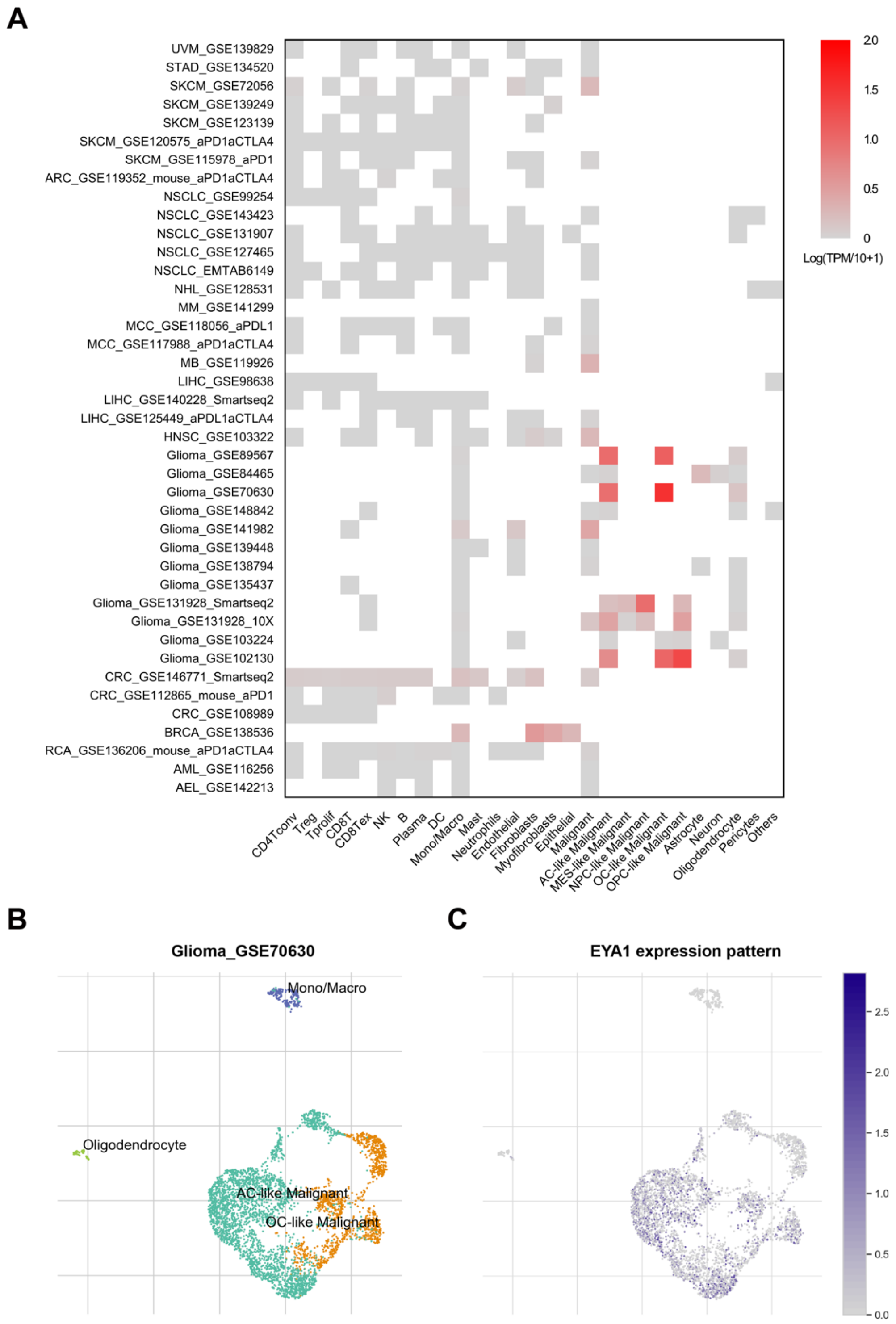

Figure S1. EYA1 is overexpressed in the malignant cells in glioma

A, Pan-cancer analysis of EYA1 mRNA level in single-cell resolution reveals that EYA1 was specifically expressed in glioma malignant cells but not immune cells.

B-C, Expression pattern analysis of EYA1 in GSE70630 dataset. Cell types were clustered (B) and EYA1 expression was shown (C).

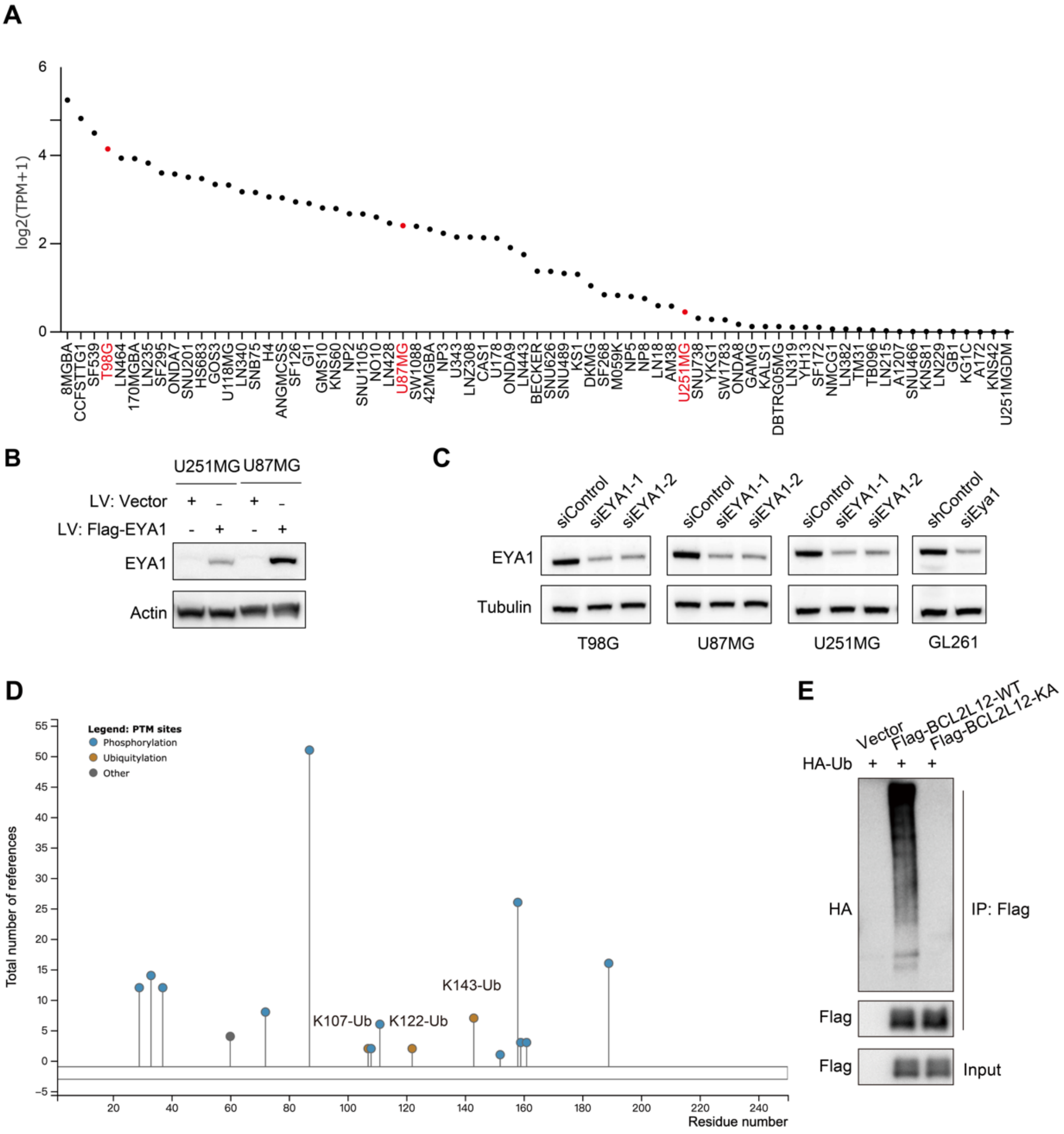

Figure S2. EYA1 expression pattern in glioma cells, overexpression and knockdown validation, and ubiquitination sites of BCL2L12

A, Expression analysis of EYA1 mRNA in different glioma cell lines from CCLE.

B-C, Immunoblotting validation of EYA1 overexpression in U87MG and U251MG (B) and knockdown in T98G, U87MG, U251MG, and GL261 (C).

D, Known ubiquitination sites in BCL2L12 in PhosphoSitePlus database.

E, Ubiquitination assay showing the ubiquitination level in wild-type and mutant BCL2L12.

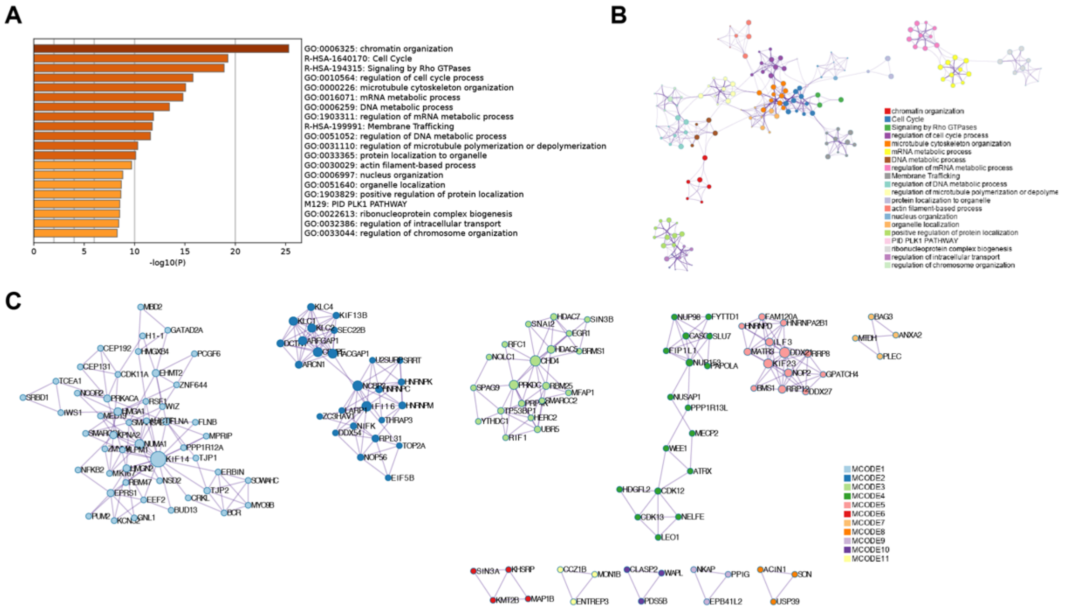

Figure S3. Enrichment analysis and protein-protein interaction network analysis of the phosphorylated proteins influenced by EYA1 overexpression

A, Enrichment analysis of the phosphorylated proteins influenced by EYA1 overexpression.

B, Clustering of the phosphorylated proteins influenced by EYA1 overexpression.

C, Protein-protein interaction network analysis of the phosphorylated proteins influenced by EYA1 overexpression.

A

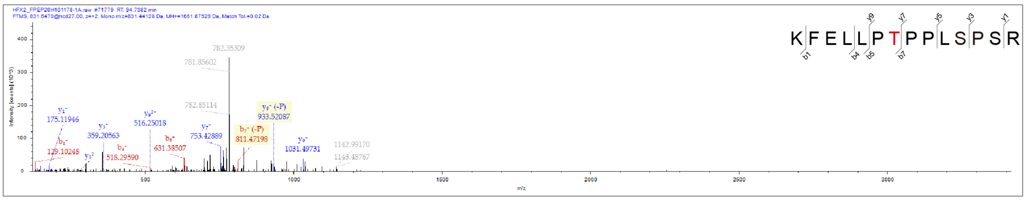

B

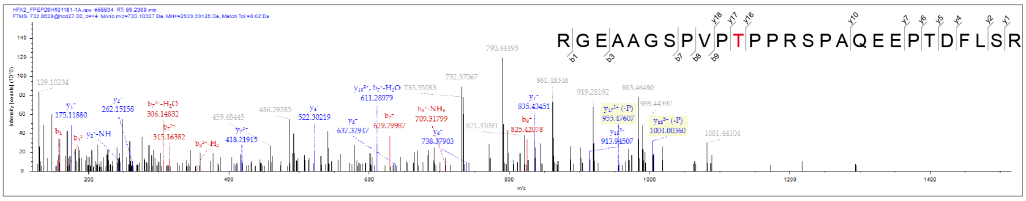

Figure S4. Representative phosphorylated peptides

Mass spectrometry of the phosphor-peptides of p-T58 in MYC (A) and p-T33 in BCL2L12 (B). Phosphorylated sites were highlighted by red.

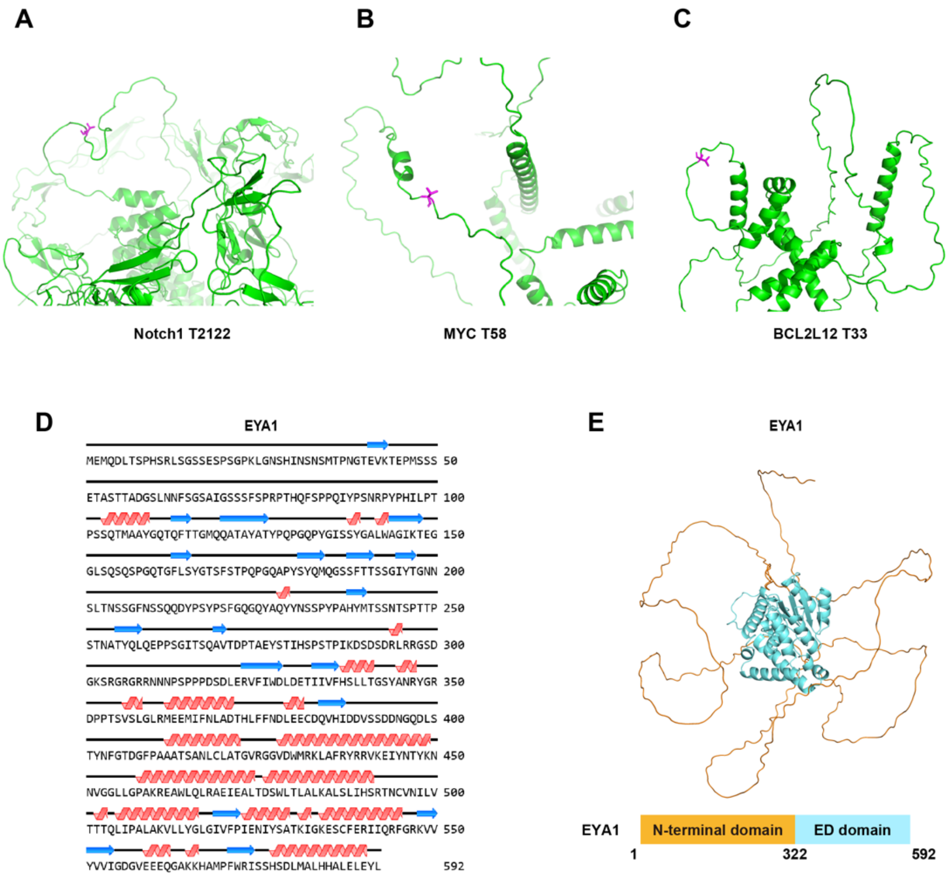

Figure S5. Predicted structure of EYA1 and its potential substrates

A-C, 3D structures of Notch1, MYC, and BCL2L12 were predicted by AlphaFold2 webserver. The potential dephosphorylation sites on these proteins by EYA1 were highlighted by pink.

D, Secondary structure analysis of EYA1 by PSIPRED webserver.

E, 3D structures of EYA1 was predicted by AlphaFold2 webserver. The flexible N-terminal domain and stable C-terminal ED domain were presented as indicated.

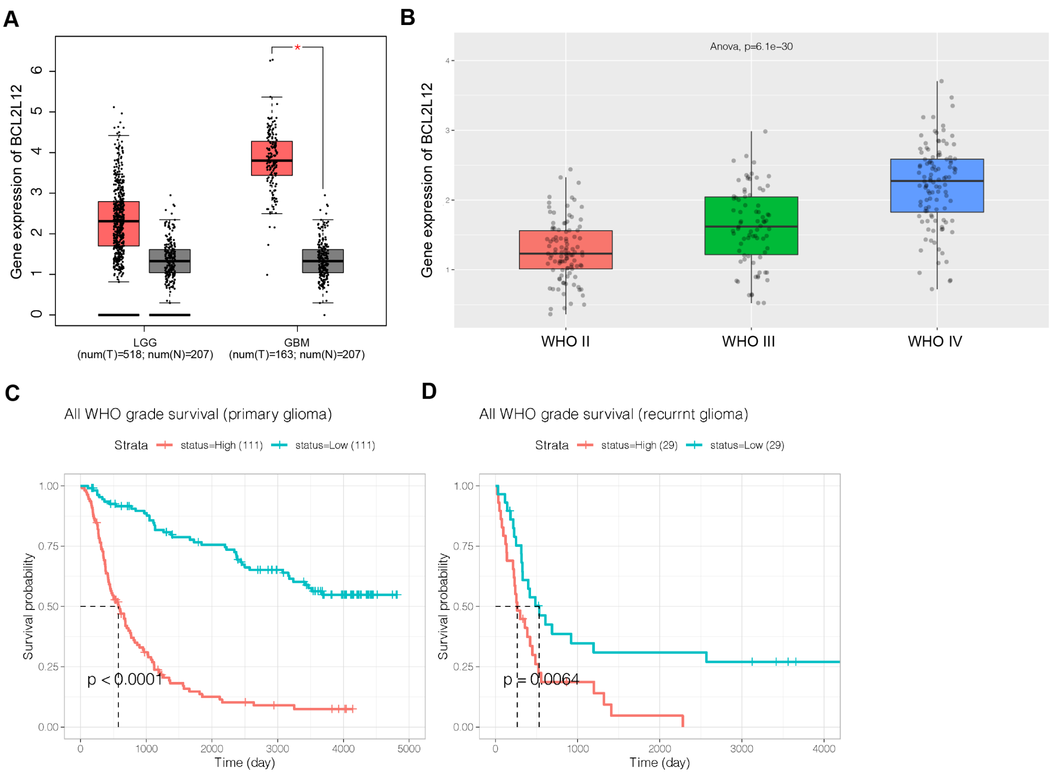

Figure S6. BCL2L12 is overexpressed in glioma and correlates with poor patient survival

A, Compared with normal brain tissues, BCL2L12 mRNA is upregulated in lower grade glioma (LGG) and glioblastoma (GBM) in TCGA dataset.

B, BCL2L12 expression level is increased with glioma malignancy in CGGA dataset.

C-D, BCL2L12 correlates with poor patient survival in both primary (C) and recurrent (D) glioma in CGGA dataset.
